# Accomplishing High‐Performance Organic Solar Sub‐Modules (≈55 cm^2^) with >16% Efficiency by Controlling the Aggregation of an Engineered Non‐Fullerene Acceptor

**DOI:** 10.1002/advs.202404997

**Published:** 2024-06-18

**Authors:** Thavamani Gokulnath, Hyerin Kim, Kakaraparthi Kranthiraja, Bo Hyeon Cho, Ho‐Yeol Park, Jesung Jee, Young Yong Kim, Jinhwan Yoon, Sung‐Ho Jin

**Affiliations:** ^1^ Department of Chemistry Education Graduate Department of Chemical Materials Institute for Plastic Information and Energy Materials Sustainable Utilization of Photovoltaic Energy Research Center (ERC) Pusan National University Busandaehakro 63‐2 Busan 46241 Republic of Korea; ^2^ Centre for Material Science Department of Chemistry and Physics Queensland University of Technology Brisbane 4000 Australia; ^3^ Beamline Division Pohang Accelerator Laboratory Pohang University of Science and Technology Pohang 37673 Republic of Korea

**Keywords:** controlled aggregation, environmentally benign solvents, high PCEs, non‐fullerene acceptor, solar sub‐modules

## Abstract

The fabrication of environmentally benign, solvent‐processed, efficient, organic photovoltaic sub‐modules remains challenging due to the rapid aggregation of the current high performance non‐fullerene acceptors (NFAs). In this regard, design of new NFAs capable of achieving optimal aggregation in large‐area organic photovoltaic modules has not been realized. Here, an NFA named BTA‐HD‐Rh is synthesized with longer (hexyl‐decyl) side chains that exhibit good solubility and optimal aggregation. Interestingly, integrating a minute amount of new NFA (BTA‐HD‐Rh) into the PM6:L8‐BO system enables the improved solubility in halogen‐free solvents (*o*‐xylene:carbon disulfide (*O*‐XY:CS_2_)) with controlled aggregation is found. Then solar sub‐modules are fabricated at ambient condition (temperature at 25 ± 3 °C and humidity: 30–45%). Ultimately, the champion 55 cm^2^ sub‐modules achieve exciting efficiency of >16% in *O*‐XY:CS_2_ solvents, which is the highest PCE reported for sub‐modules. Notably, the highest efficiency of BTA‐HD‐Rh doped PM6:L8‐BO is very well correlated with high miscibility with low Flory‐Huggins parameter (0.372), well‐defined nanoscale morphology, and high charge transport. This study demonstrates that a careful choice of side chain engineering for an NFA offers fascinating features that control the overall aggregation of active layer, which results in superior sub‐module performance with environmental‐friendly solvents.

## Graphical abstract

A remarkable PCE of >16% is accomplished through enhanced morphology in 55 cm^2^ sub‐modules fabricated using environmentally benign non‐halogen solvents (*O*‐XY:CS_2_) and longer side‐chains (hexyl‐decyl) assisted new NFA (BTA‐HD‐Rh). This notable performance is attributed to an improved nano‐scale morphology, intra‐ and intermolecular interactions, lesser recombinations, and film aggregations. In addition, optimized devices have operational stabilities.

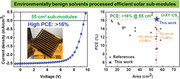

## 1 Introduction

Over the past decade, research on organic solar cells (OSCs) has advanced, leading to an increased photovoltaic performance. Thanks to their superior features including affordability, resilience, and ease of large area manufacturing, OSCs can be employed in diverse array of applications as an efficient alternative to inorganic solar cells. In addition, ternary blend OSCs offer other benefits, including controllable nano‐morphology, tunable bandgaps, strong and broad absorption in a wide range, and high charge carrier transport properties that can result in improved power conversion efficiency (PCE).^[^ ^1^, ^2^, ^3^ ^]^ Currently, with the optimization of the device fabrication process and innovation of organic materials (polymer donor and non‐fullerene acceptor (NFA)), the small‐area ternary blend OSCs have reached PCEs of >19%.^[^ ^4^, ^5^, ^6^ ^]^ However, these high efficiencies were recorded in laboratory scale devices. Therefore, it is substantially important to extend this technology to enable solution processing of active layer materials at room temperature (RT) to facilitate the fabrication of solar sub‐modules, contributing to the development of commercial electronic devices.^[^ ^7^, ^8^, ^9^ ^]^ Presently, a certified PCE of 12% has been recorded for a 58 cm^2^ solar sub‐module,^[^ ^8^ ^]^ which is inferior to the peak performance achieved in the lab scale (>19%). Compared to small‐area active layer films, the quality of sub‐module active layer films deposited on large substrates is poor. This is primarily due to the challenges in achieving sufficient solubility active layer components, miscibility, processability, and rough surface of large substrates.^[^ ^9^, ^10^, ^11^ ^]^ Unlike, small area devices that are spin coated in a glove box, large area devices fabricated at RT face challenges as excess aggregation, oversized domains of active layer materials due to the long‐evaporation times of processing solvents. Besides, it is also a concern to develop the large area sub‐modules with toxicity of processing solvents. Therefore, the ability to use environmentally benign solvents in the production of efficient and reproducible active layer materials is of considerable importance in bridging the gap between small‐area and sub‐module devices.

Although significant efforts have been made in tailoring the molecular structure of photoactive materials, limited research has been done on the challenges such as thickness sensitive PCEs, film aggregation and phase separation in sub‐modules to that of small‐area devices that are being developed in research labs. Thus it has become increasingly challenging to manufacture homogeneous, uniform active layer films with the desired nano‐morphology for excellent exciton generation and transport properties in sub‐modules with high PCEs.^[^ ^12^, ^13^, ^14^, ^15^, ^16^, ^17^ ^]^

The most common laboratory approaches in recent times are bulk‐heterojunction (BHJ) techniques, which yield small‐area devices with high PCEs. Nevertheless, excessive aggregation of BHJ active layer blends led to limited PCEs for sub‐module devices. It has been demonstrated that modulating the alkyl chain on NFAs has a notable impact on fine‐tuning the overall aggregation of the active layer morphology. Furthermore, through NFA engineering, it appears that domain size has been successfully regulated to improve the hole transfer efficiency by suppressing the undesired recombination. Incidentally, a maximum PCE of >14% for 25, 18, and 58.5 cm^2^ has been achieved for binary and ternary blend devices, respectively.^[^ ^18^, ^19^, ^20^ ^]^ Moreover, the effect of NFA side chain engineering has further contributed to extending the halogen‐free solvent processing for large area devices.^[^ ^21^, ^22^ ^]^ Recently, our group reported PCEs of 13.88% and 12.20% with 55 cm^2^ sub‐modules processed using halogen‐free and halogen solvents with different active layer (ternary and quaternary blend) systems.^[^ ^23^, ^24^ ^]^ Based on the above results, we hypothesize that molecular structural engineering (side chain modulation) of NFAs has significantly alter the solubility, blend miscibility, and aggregation in active layer. All these factors are expected to contribute to enhance the PCEs of solar sub‐modules. However, only a limited examples have been demonstrated the use of environmentally benign solvents to produce ternary blend sub‐module with moderate PCEs.

In the present work, we synthesized a new NFA (BTA‐HD‐Rh) with hexyl decyl side chain to improve the solubility and to control the aggregation. Interestingly, inclusion of small amount of BTA‐HD‐Rh into PM6:L8‐BO blend reduced the excess aggregation arises from the L8‐BO in *o*‐xylene:carbon disulfide (*O*‐XY:CS_2_), v/v = 0.6:0.4. Owing to the excellent solution processability, improved miscibility among the active layer components and controlled active layer film aggregations, bar‐coated ternary blend sub‐module (55 cm^2^) devices exhibited prominent PCEs of >16% and *V*_oc_, *J_sc_*, and good FF values of 9.49 V, 2.50 mA cm^−2^, and 67.36%, which should be the highest PCE of solar sub‐modules (Table S1, Supporting Information). Overall, this study has provided a decent example of a high performance sub‐module organic photovoltaics through a molecular engineering of NFA.

## 2 Results and Discussion

### 2.1 New NFA Synthesis and Characterization

**Figure 1:**
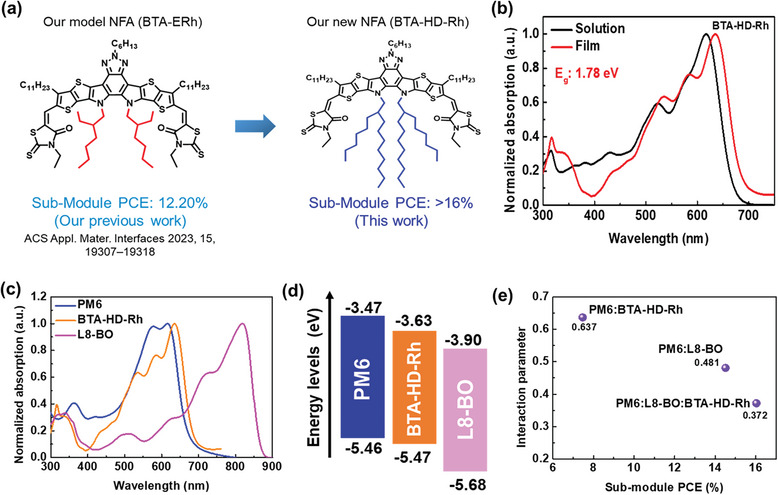
a) Our model and newly synthesized NFAs (BTA‐ERh^[^ ^24^ ^]^ and BTA‐HD‐Rh). b) UV–vis absorption spectra of BTA‐HD‐Rh. c) UV–vis film absorption spectra and d) energy levels diagram of PM6, L8‐BO, BTA‐HD‐Rh used in this study. e) Flory‐Huggins interaction parameters (χ) of PM6:BTA‐HD‐Rh, PM6:L8‐BO, and PM6:L8‐BO:BTA‐HD‐Rh blends.

**Figure** **1**a shows our model and newly synthesized NFAs (BTA‐ERh^[^ ^24^ ^]^ and BTA‐HD‐Rh). Figure S1 (Supporting Information) displays the molecular structure of PM6 polymer donor, L8‐BO, Y7‐BO, Y6‐BO, and BTP‐eC9 NFAs structure. The synthetic routes for BTA‐HD‐Rh are presented in Scheme S1 (Supporting Information). The BTA‐HD‐Rh was confirmed by ^1^H, ^13^C NMR, and mass spectra (Figure S2a,b, Supporting Information). Owing to the long side chains presented in molecular structure, BTA‐HD‐Rh has superior solubility in common organic solvents. The basic properties of BTA‐HD‐Rh are detailed in Table S2 (Supporting Information). The thermal properties of BTA‐HD‐Rh were studied by thermal gravimetric analysis (TGA) (Figure S3a, Supporting Information). The decomposition temperature of BTA‐HD‐Rh is 373 °C, indicating excellent thermal stability compared to our model NFA (Table S2, Supporting Information). A crystalline peak location at 104 °C was revealed by differential scanning calorimetry (DSC) of BTA‐HD‐Rh (Figure S3b, Supporting Information), indicating that the addition of BTA‐HD‐Rh enhanced active layer molecular aggregation and device stability.

The absorption properties of BTA‐HD‐Rh can be seen in the UV–vis absorption spectra. Figure 1b shows that the acceptor has a nearly comparable absorption, with the absorption maximum located at 618 nm. Because of the molecular packing, the normalized film absorption of BTA‐HD‐Rh shows a significant redshift (637 nm) as compared to the solution.^[^ ^22^, ^25^ ^]^ Additionally, Figure 1c and Figure S4a (Supporting Information) show the film absorption of acceptor complements with the PM6 and L8‐BO. This complimentary absorption of the multiple active components should be beneficial for exciton formation and charge extraction properties.^[^ ^24^, ^26^ ^]^ The film absorption coefficient at the maximum peaks for BTA‐HD‐Rh is 2.8 × 10^−2^ cm^−1^ (Figure S4b, Supporting Information), which was greater than that of L8‐BO. Also, the optimized ternary blend absorption co‐efficient value was greater than that of the optimized binary blend film (Figure S4a, Supporting Information), which would utilize more solar photons and enhance the *J_sc_*_._^[^ ^24^ ^]^ The optical bandgap (*E*_g_) of BTA‐HD‐Rh was estimated to be 1.78 eV (Table S2, Supporting Information). BTA‐HD‐Rh energy levels were estimated by cyclic voltammetry, as shown in Figure S5 (Supporting Information). For BTA‐HD‐Rh, the calculated highest occupied molecular orbital (HOMO)/the lowest unoccupied molecular orbital (LUMO) energy levels were −5.47/−3.63 eV, respectively (Figure 1d). As indicated in Figure 1d, cascade energy levels alignment forms between the HOMOs and the LUMOs of these active layer components. Notably, cascading energy levels increase device performance by reducing the charge transfer barrier and promoting charge transfer/collecting properties.^[^ ^27a^ ^]^

### 2.2 Aggregation Properties of Binary and Ternary Blends

**Figure 2:**
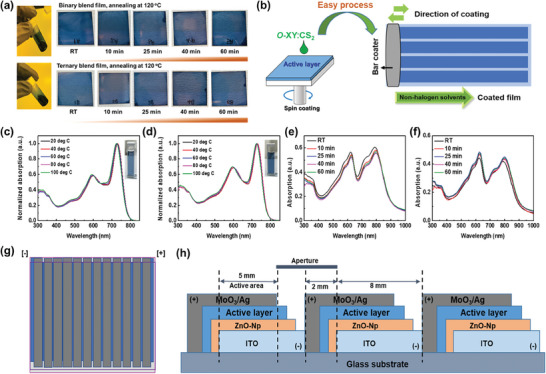
a) Photograph images of binary and ternary blend films annealed at 120 °C at different time intervals. b) Schematic structure of spin and bar coated films. Temperature dependent solution UV–vis absorption spectra of c) binary and d) ternary blends. UV–vis absorption spectra of e) binary and f) ternary blend films annealed at 120 °C at different time intervals. g) Top view and h) schematic diagram of the sub‐module fabrication design (11 cells connected in series).

We performed the temperature dependent absorption spectroscopy for binary and ternary blend solution and films to better understand the aggregation behavior. The preparation of binary and ternary blend film photographs is shown in **Figure** **2**a. And, Figure 2b displays schematic structure of spin and bar coated films. As shown in Figure 2c,d, upon raising the temperature the absorption spectra of binary blend (PM6:L8‐BO) solution showed slight variation in ICT band, while ternary blend (PM6:L8‐BO:BTA‐HD‐Rh) solution spectra remained unchanged. Next, Figure 2e,f shows the binary and ternary blend film temperature dependent absorption spectra. Interestingly, binary blend film showed reduced aggregation upon heating, while ternary blend showed less aggregation over the annealed ternary blend film. This clearly shows that addition of small amount of new NFA into the binary blend not only improving the solubility of ternary blend but also restricts the excess aggregations.^[^ ^27b^ ^]^

### 2.3 Photovoltaic Properties

**Figure 3:**
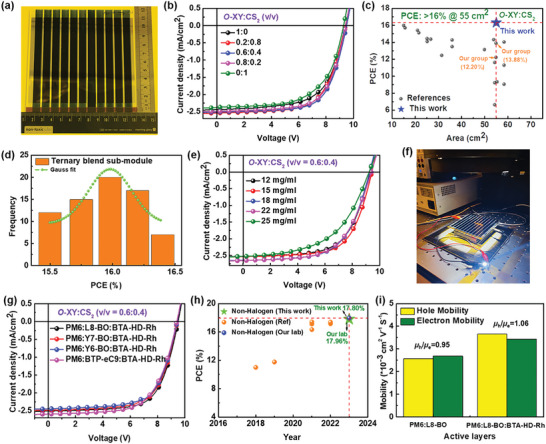
a) Image of sub‐module device. b) *J–V* curves of the optimized ternary blend (PM6:L8‐BO:BTA‐HD‐Rh) sub‐module with *O*‐XY:CS_2_ solvent ratios. c) PCEs of solar sub‐module with areas >20 cm^2^ (Table S1, Supporting Information). d) Histogram PCEs of ternary blend sub‐modules. e) Best *J–V* curves of the ternary sub‐module with blend concentrations. f) The image of white LED lighting powered by organic sub‐module. g) Best *J–V* curves of *O*‐XY:CS_2_ processed sub‐modules with different NFAs. h) PCEs of air‐processed small‐area devices using non‐halogen solvents (Table S9, Supporting Information). i) Charge carrier mobilities of optimized binary and ternary devices.

In order to explore the effect of hexyl‐decyl side chains on the solar sub‐module photovoltaic performance of NFA (BTA‐HD‐Rh), sub‐module devices were fabricated by a bar coater under laboratory conditions.^[^ ^23^, ^24^ ^]^ A top view, schematic figure of the sub‐module, and its fabrication are shown in Figures 2g,h and S6 (Supporting Information). Photographs of sub‐module and bar coater equipment images are shown in **Figures** **3**a and S7 (Supporting Information). The successful use of environmentally friendly solution‐processing techniques for large‐scale module printing is essential to the commercialization of organic solar technology. However, aggregation effects and strong bimolecular recombination properties frequently occur in environmentally benign solvents processed sub‐modules and reduce FFs, PCEs.^[^ ^19^, ^20^, ^22^, ^23^, ^24^, ^28^ ^]^ In this regard, BTA‐HD‐Rh was added into binary blend film (PM6:L8‐BO) to create efficient 55 cm^2^ sub‐modules with well‐constructed, pinhole‐free BHJ morphology and fewer recombination with environmentally benign non‐halogen solvents. A detailed explanation of the sub‐module device fabrication is provided in Supporting Information. The *J*–*V* curves of the champion ternary blend sub‐module and their device parameters are displayed in Figure 3b and **Table** **1**. The FF's and PCE's were significantly lower for the single solvent processed devices. After well optimizations, the *O*‐XY:CS_2_ (v/v = 0.6:0.4) processed device based on the PM6:L8‐BO:BTA‐HD‐Rh blend demonstrated remarkable device performances (*V*_oc_ of 9.49 V, *J*_sc_ of 2.50 mA cm^−2^, and good FF of 67.36%), which resulted in a PCE of 16.03% for a 170 nm active layer film with a geometric fill factor of 62.5%. Notably, the PCE of the BTA‐HD‐Rh added device was >16%, which was among the highest reported for non‐halogen solvents processed solar sub‐module devices (Figure 3c; Table S1, Supporting Information). Table S3 (Supporting Information) displays the photovoltaic parameters that were discovered throughout the sub‐module optimization process. Also, the histogram depicted in Figure 3d shows the distribution the PCEs of ternary blend devices, confirms the superiority and reliability of the sub‐modules. In addition, devices with different blend concentrations were evaluated. The PCEs for ternary blend sub‐modules were consistently >12%, for example, 14.80% at 12 mg mL^−1^, 16.03% at 15 mg mL^−1^, 15.17% at 18 mg mL^−1^, 14.88% at 22 mg mL^−1^ and 12.27% at 25 mg mL^−1^ (Figure 3e; Table S4, Supporting Information). These results demonstrate that PM6:L8‐BO:BTA‐HD‐Rh is an excellent candidate for sub‐module device manufacture. Binary blend (PM6:BTA‐HD‐Rh and PM6:L8‐BO) sub‐module device PCEs are shown in Figure S8a and Table S5 (Supporting Information). These devices have poor exciton formation and charge transfer properties, which were confirmed by the lowest FF and *J*_sc_ values. In addition, the halogen solvent processed sub‐module devices exhibited a good PCE of 14.42% and *V*_oc_, *J*_sc_, and FF values of 9.41 V, 2.43 mA cm^−2^, 62.88%, respectively (Figure S8b, Supporting Information). Moreover, when the organic sub‐module was illuminated with 1 sun intensity light, bright emission was observed from a white light‐emitting diode (LED), indicating successful sub‐module operation (Figure 3f), suggesting promise for commercialization.

**Table 1:** Detailed sub‐module photovoltaic parameters of processed by environmentally benign non‐halogen solvents ratio (v/v) of ternary blend active layers (PM6:L8‐BO:BTA‐HD‐Rh) under an illumination of AM 1.5G at 100 mW cm^‒2^.

*O*‐XY:CS_2_ ^a)^	*V*_oc_ [V]	*J*_sc_ [mA cm^−2^]	FF [%]	PCE^b)^ [%]
1:0	9.41 (9.09 ± 0.32)	2.43 (2.19 ± 0.24)	62.88 (62.38 ± 0.5)	14.42 (14.20 ± 0.22)
0.2:0.8	9.42 (9.27 ± 0.15)	2.53 (2.35 ± 0.18)	65.07 (64.47 ± 0.6)	15.55 (15.30 ± 0.25)
0.6:0.4	9.49 (9.37 ± 0.12)	2.50 (2.37 ± 0.13)	67.36 (67.06 ± 0.3)	16.03 (15.93 ± 0.10)
0.8:0.2	9.43 (9.16 ± 0.27)	2.48 (2.21 ± 0.27)	66.60 (65.90 ± 0.7)	15.62 (15.24 ± 0.38)
0:1	9.28 (9.10 ± 0.18)	2.37 (2.14 ± 0.23)	60.96 (60.76 ± 0.2)	13.42 (13.15 ± 0.27)

^a)^ Active layer films were annealed at 120 °C/20 min;

^b)^ An average of 15 sub‐modules were evaluated.

The best practical choices for building sub‐module devices were determined to be among the different NFAs examining ternary blend systems. The best *J*–*V* graphs are presented in Figure 3g, and Table S6 (Supporting Information) has detailed values for the photovoltaic parameters. In particular, maximum PCEs of >15% were achieved by these ternary blend systems with outstanding FFs values of >60%; all were processed the *O*‐XY:CS_2_ solvents system. Notably, when BTA‐HD‐Rh was added to both ternary blends, we obtained smooth film surfaces and good interpenetrating morphologies, which are known to increase sub‐module FFs and PCEs. This result shows that the morphologies of ternary blend film can homogeneously form optimal BHJ morphology even in sub‐module devices. This reproducible morphology of the BTA‐HD‐Rh added ternary blend film is one of the most essential factors that decrease the FF loss throughout the cell to module upscaling process.^[^ ^14^ ^]^ Usually, FF loss can be affected by inhomogeneous morphology and excessive aggregation, which are produced more often as the active area rises.^[^ ^14^ ^]^ Therefore, it is vital to develop active materials that can form the optimal BHJ morphology in organic sub‐modules to ensure the commercialization of solar technology.

We also analyzed and fabricated small‐area devices with binary and ternary blend combinations to support our sub‐module results. The supporting information section provides detailed fabrication procedures for small‐area devices. The optimal PM6:L8‐BO binary device had a *V*_oc_ of 0.85 V, *J*_sc_ of 26.12 mA cm^−2^, an FF 71.26%, and a PCE 16.04% (Figure S9a and Table S7, Supporting Information). After a series of optimizations (Table S8, Supporting Information) and introducing 0.2 weight ratio (wt. ratio) BTA‐HD‐Rh into the binary blend, the resultant ternary small‐area device provided a good PCE of 17.80% and improved *V*_oc_, *J*_sc,_ and FF values of 0.86 V, 27.54 mA cm^−2^, and 75.08%, respectively. This PCE is one of the highest reported PCEs of ambient processed small‐area devices^[^ ^23^ ^]^ (Figure 3h; Table S9, Supporting Information). Additionally, steady‐state measurements of optimized binary and ternary devices produced PCEs of 16.01% and 17.17% (Figure S10, Supporting Information), which concurred with *J–* *V* results. In Figure S9b (Supporting Information) displays the external quantum efficiency (EQE) spectra of the optimized devices. The calculated integrated *J_sc_* values correlated with *J–* *V* data. Notably, efficiency of ternary blend devices was shown to yield greater FF and EQE values compared to binary blend devices. The maximum EQE measurements show that it can be partially ascribed to more effective charge and energy transfer between the active layer.^[^ ^24^ ^]^

The effect of BTA‐HD‐Rh loadings on device hole (*µ*_h_) and electron (*µ*_e_) mobilities was examined using the space‐charge‐limited current (SCLC) method^[^ ^3^, ^24^ ^]^ (Figure 3i; Figure S11, Supporting Information). The optimized blend films had dramatically different *µ*_h_ and *µ*_e_ values (Table S10, Supporting Information). The *µ*_h_ and *µ*_e_ values of optimized BTA‐HD‐Rh added ternary blend devices were greatly increased to 3.65 × 10^−3^ and 3.43 × 10^−3^ cm^2^ V^−1^ s^−1^, respectively. Also, adding BTA‐HD‐Rh to L8‐BO improved the *µ*_e_ values. Moreover, our model NFA (BTA‐ERh) had a lower *µ*_e_ value than the pristine BTA‐HD‐Rh, suggesting effective charge production. Furthermore, ternary devices well‐balanced charge carrier transport (1.06), which support enhanced *J_sc_* and FF values.

### 2.4 Active Layer Morphology Investigation

**Figure 4:**
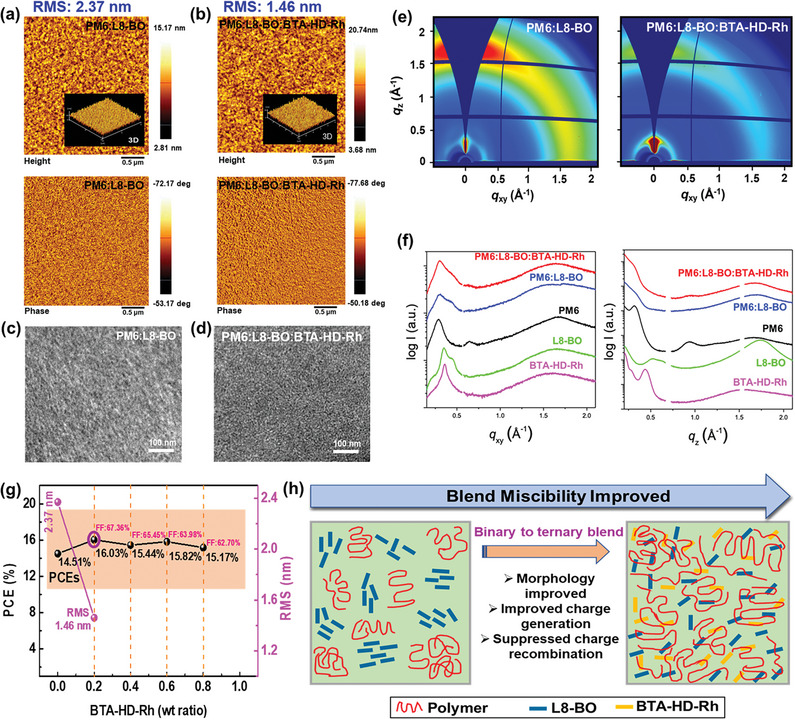
Morphological a,b) AFM, c,d) TEM images of optimized blend films. e) 2D, f) 1D (IP and OOP directions)‐GIWAXS images of optimized films. g) PCE versus film roughness values of BTA‐HD‐Rh different weight ratios into PM6:L8‐BO blend. h) Schematic illustration of the BHJ morphologies in terms of BTA‐HD‐Rh content.

The optimized blend film morphologies were investigated by atomic force microscopy (AFM) and transmission electron microscopy (TEM).^[^ ^22^, ^23^ ^]^ Blend films containing BTA‐HD‐Rh exhibited a nano‐fibrillar network, which is favorable to efficient exciton dissociation.^[^ ^21^, ^29^, ^30^ ^]^ It is interesting to note that after adding BTA‐HD‐Rh to the PM6:L8‐BO blend, the root‐mean‐square (RMS) surface film roughness decreased from 2.37 to 1.46 nm (**Figure** **4**a,b), suggesting that BTA‐HD‐Rh were improved morphology of the optimal ternary blend film. Furthermore, to evaluate the effects of BTA‐HD‐Rh on the domain morphology of L8‐BO, AFM images of the BTA‐HD‐Rh, L8‐BO and L8‐BO:BTA‐HD‐Rh films were displayed in Figure S12 (Supporting Information). These results showed longer side chains affected acceptor domains in *O*‐XY:CS_2_ processed blend films, which is confirmed by TEM analysis. Here, Figure 4c shows how the binary blend intimate blending produced bigger domains, which may have limited the development of the blended films interpenetrating morphology of BTA‐HD‐Rh and reduced the yields of charge transfer and geminate recombination.^[^ ^31^ ^]^ On the other hand, upon adding BTA‐HD‐Rh to the PM6:L8‐BO blend, smaller domains with interpenetrating network topology (Figure 4d) were seen. This might lead to improved exciton dissociation and fewer recombinations with noticeable increases in FF and *J_sc_*. More importantly, sub‐module blend films exhibited similar morphology trends, and this was confirmed by AFM analysis (Figure S13, Supporting Information). The above‐mentioned results indicated that BTA‐HD‐Rh is a promising candidate for printed electronics due to ideal BHJ morphology.

Additionally, grazing incidence wide‐angle X‐ray scattering (GIWAXS) measurements were performed to observe the morphology of the PM6, L8‐BO, BTA‐HD‐Rh, and binary and ternary blends. We reveal that L8‐BO and BTA‐HD‐Rh have a highly crystalline nature, while PM6 shows a randomly oriented layered structure (Figure S14, Supporting Information). In Figure 4e shows 2D‐GIWAXS images for PM6:L8‐BO and PM6:L8‐BO:BTA‐HD‐Rh. For the 2D‐GIWAXS pattern for PM6:L8‐BO, we found some characteristics reflections of the crystalline L8‐BO are still remained near the small *q* region, while it is fully disappeared in the 2D pattern for PM6:L8‐BO:BTA‐HD‐Rh. 1D profiles extracted from the 2D patterns also indicate the improved miscibility of the ternary blend film. Further, water contact angle study results on miscibility are in line with the GIWAXS results (vide infra). As shown in Figure 4f, sharp reflections of the L8‐BO and BTA‐HD‐Rh are not significantly appeared in both in‐plane (IP) and out‐of‐plane (OOP) profiles of the ternary blend system, while shoulder peak attributed by crystalline L8‐BO is appeared in PM6:L8‐BO. These results clearly indicate the BTA‐HD‐Rh improves the blend miscibility, leading an enhancement in charge transport properties and suppression of the excessive aggregation in ternary blend films.

Further, the molecular behavior of the optimized blend film was investigated by energy‐dispersive X‐ray spectroscopy (EDS) mapping. Polymer and acceptor distributions were determined by EDS mapping. The images of the S, O, and F atomic signals collected are shown in Figure S15 (Supporting Information). S atom distribution can be used to examine the behavior of the active layers, which compose a large component of the molecular structure. Notably, F, O, and S were uniformly distributed in the ternary blend BHJ layer.

In Figure 4g displays the PCEs and FFs remained at >15% and >62% as the content of BTA‐HD‐Rh improved from 0.2 to 0.8 wt. ratio, indicating BTA‐HD‐Rh is a BHJ morphology regulator. Also, BTA‐HD‐Rh improved the blend miscibility and molecular packing morphology of the ternary blend film (Figure 4h), which is known to increase exciton dissociation, charge transport properties, and support suppressed recombinations, and thus, increase FFs and PCEs. Figure S16a (Supporting Information) shows photographic images of binary and ternary blend module films. The PM6:L8‐BO films showed more regions of aggregation after bar‐coating. After introducing BTA‐HD‐Rh into PM6:L8‐BO blend, ternary blend sub‐module film morphology clarity improved without aggregation, which is confirmed by AFM and optical microscope analysis (Figures S16b and S17, Supporting Information). The improved active layer film morphology was ascribed to better solubility and blend miscibilities, which are more discussed below.

Phase separation in active layer blend films has been observed to be impacted by variations in surface energy (*γ*).^[^ ^26^, ^32^ ^]^ According to Figure S18 and Table S11 (Supporting Information), the *γ_s_* values for PM6, L8‐BO, BTA‐HD‐Rh, and L8‐BO:BTA‐HD‐Rh were 28.9, 32.5, 31.1, and 33.2 mJ m^−2^, respectively. Although the *γ_s_* values of NFA films were considerably similar, it is possible that NFA formed a well‐mixed phase, which led to more efficient intramolecular charge transfer (ICT). Further, the Flory‐Huggins interaction parameter (χ) was estimated from *γ_s_* values in order to have a better understanding of the miscibility of photoactive materials in the active layer.^[^ ^32^, ^33^ ^]^ The determined χ values for PM6 and L8‐BO, PM6 and BTA‐HD‐Rh, PM6:L8‐BO and BTA‐HD‐Rh were 0.481, 0.637, and 0.372, respectively (Figure 1e). When BTA‐HD‐Rh was added, the interaction value for PM6:L8‐BO significantly decreased from 0.481 to 0.372, suggesting that the addition of BTA‐HD‐Rh improved the miscibility between the active layers of ternary blends. Furthermore, it is possible that the active layers specific miscibilities prevented unwanted mixing during the BHJ. Additionally, the relative interaction parameters of BTA‐HD‐Rh enhanced miscibility and led to the establishment of a suitable phase distribution during the production of ternary blend active layers. These results suggest that phase separations are more likely to occur when the donor and acceptor are relatively miscible. These results demonstrate the presence of BTA‐HD‐Rh well‐balanced miscibility and increased morphology of ternary blend films. Thus, support to improve FF values without excessive aggregations.

### 2.5 Carrier Dynamic Properties

The connection between photocurrent (*J*_ph_) and effective voltage (*V*_eff_) was studied to investigate the exciton dissociations of optimized devices.^[^ ^23^ ^]^ Figure S19a (Supporting Information) shows that the optimized devices saturation photocurrent densities at 1 V were *J*_ph_ and *J*_sat_, demonstrating efficient charge transfer and collection in the active layers. For binary (PM6:L8‐BO) and ternary (PM6:L8‐BO:BTA‐HD‐Rh) devices, the maximal exciton generation rates (*G*_max_) were 1.25 × 10^28^ and 1.39 × 10^28^ m^−3^ s^−1^, respectively. Furthermore, compared to the binary device (92.10%), the ternary device (97.07%) showed a higher exciton dissociation probability (Table S12, Supporting Information), suggesting a more efficient exciton dissociation at the active layer interfaces, which was confirmed by higher *J*_sc_’s and FF's. Furthermore, Figure S19b (Supporting Information) shows the linear plots of light intensity (LI) against *J*_sc_ [*P*_light_ (*J*_sc_ ∝ *P*_light_^α^)] demonstrated the predominance of bimolecular recombination in the ternary device (0.96 *kT q* ^−1^). For binary and ternary devices, the slopes (α) of the LI versus *V*_oc_ [*V*_oc_ ∝ n(*k*T q^−1^)ln(*P*_light_)] plots were 1.15 and 1.07 *kT q* ^−1^, respectively (Figure S19c, Supporting Information). Importantly, the lower slopes for PM6:L8‐BO:BTA‐HD‐Rh would contribute to decreased trap‐assisted recombinations. According to these findings, the ternary device offered greater PCEs, less morphological traps, and more efficient charge transfer. Moreover, at low light intensities, ternary devices exhibited higher FFs (Figure S19d, Supporting Information), while binary devices exhibited noticeably lower FFs at high light intensities. These findings imply that binary devices induced free carrier recombination and produced FF losses as the number of free carriers rose with *P*_light_, indicating that the strong ICT features of BTA‐HD‐Rh enhanced charge transport paths. Additionally, the ternary device (2926 cm^2^) has a lower charge transport resistance (*R*_CT_) than the binary device (8161 cm^2^), as revealed by impedance spectra (Figure S20, Supporting Information).^[^ ^24^, ^34^ ^]^ This would assist in decreasing charge recombinations and increase the FF and *J_sc_* values.

Exciton quenching effects were evaluated by photoluminescence (PL) spectroscopy. Normalized PL spectra of PM6:L8‐BO and PM6:L8‐BO:BTA‐HD‐Rh blend films excited at 620 nm are shown in Figure S21a (Supporting Information). BTA‐HD‐Rh quenched PM6 emission in blend films without detectable peaks. The PM6:L8‐BO:BTA‐HD‐Rh film showed more quenching at 637 nm when stimulated compared than the PM6:L8‐BO film, suggesting that the ternary blend film has better exciton dissociation efficiency (Figure S21b, Supporting Information).^[^ ^23^, ^26^ ^]^ Furthermore, PM6 and BTA‐HD‐Rh both demonstrated a significant excited peaks at 620 and 637 nm and BTA‐HD‐Rh produced an emission peak at 671 nm (Figure S21c, Supporting Information), which was strong evidence of efficient exciton dissociation and charge transfer between the active layers in efficient ternary blends. As shown in Figure S22 (Supporting Information), a time‐resolved PL (TRPL) experiment was performed to compare exciton lifespans in binary and ternary blend films. The optimized ternary film had a substantially lower exciton lifetime of ≈1.97 ns under 620 nm excitation, compared to the binary film of ≈2.37 ns. Therefore, the ternary devices had efficient energy and charge transfer processes. We also analyzed the *J–V* curves of L8‐BO, BTA‐HD‐Rh, L8‐BO:BTA‐HD‐Rh based devices. The *V*_oc_, *J*_sc_ and FF values of L8‐BO:BTA‐HD‐Rh devices were higher than those of L8‐BO and BTA‐HD‐Rh (Figure S23, Supporting Information), which highlighted efficient charge transfer occurred between the interfaces of NFAs. Moreover, for BTA‐HD‐Rh blended ternary devices, estimated trap‐state densitiy^[^ ^35^ ^]^ was 1.65 × 10^15^ cm^−3^ (Figure S24, Supporting Information). The ternary device exhibited lower trap‐state densities than the binary device. This difference could be ascribed to the improved blend films ideal morphology and increased miscibility, both of which facilitate charge carrier properties. These findings suggest that BTA‐HD‐Rh is an excellent choice for producing efficient solar sub‐module devices using non‐halogen solvents that are harmless to the environment.

### 2.6 Device Stability Analysis

For industrial applications, sub‐module active layer components need to be stable and compatible with variations in film thickness.^[^ ^24^, ^36^ ^]^ Significantly, the components of the active layer were hydrophobic both in their pristine and blend forms (Figures S18 and S25, Supporting Information). Hydrophobicity improves the environmental stabilities of optimized devices by preventing water penetration.^[^ ^37^ ^]^

**Figure 5:**
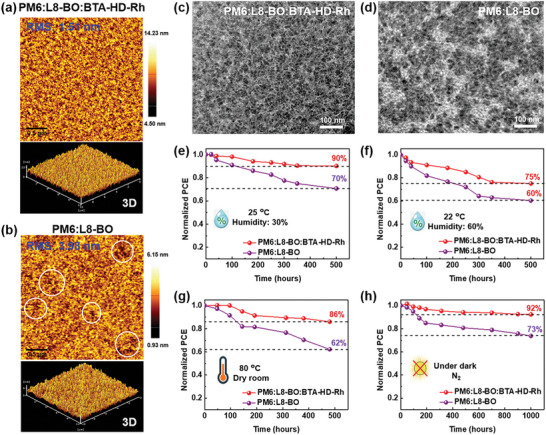
a,b) AFM, c,d) TEM morphology aging test of *O*‐XY:CS_2_ processed aged blend films. Stabilities of sub‐module optimized devices e,f) with different relative humidity in air atmosphere and g) thermal stabilities. h) In N_2_ atmosphere stability of small‐area optimized devices.

After 1000 h of continuous aging, AFM and TEM pictures of optimized active layer films were acquired to explore the connection between exciton diffusion decay and structural changes (**Figure** **5**a,b). The fibrous nanostructures in fresh binary and ternary films were distinct. Furthermore, the mean roughness of the binary blend film (PM6:L8‐BO) increased dramatically during aging, from 2.37 to 3.98 nm, and AFM images revealed an abundance of uneven aggregates. In contrast, the homogeneity and roughness of the ternary blend film (PM6:L8‐BO:BTA‐HD‐Rh) remained unchanged with fresh (1.46 nm) and aging (1.51 nm). Furthermore, TEM investigation verified that changes were minimal (Figure 5c,d), demonstrating BTA‐HD‐Rh capacity to inhibit aggregations. As a result, the intermixed morphology and stabilities of the ternary blend devices remained outstanding.

The atmospheric stabilities of ternary blend sub‐modules were >90% and >75% after 500 h with different relative humidity (RH) conditions (Figure 5e: 25 °C, RH: 30% and Figure 5f: 22 °C, RH: 60%), respectively (Figures S26 and S27, Supporting Information). Additionally, ternary blend sub‐module devices exhibited better thermal stability than binary blend sub‐module devices at 80 °C (Figure 5g; Figure S28, Supporting Information). Moreover, the stability of ternary blend sub‐modules was greater than that of binary blend sub‐modules under both conditions, and morphologic stability was also increased by improvements in the active layer structure.

For practical purposes, solar cell devices must be stable to prolonged light exposure,^[^ ^21^, ^38^ ^]^ and thus, we assessed the long‐term light soaking (LS) stability of the optimized devices under a light intensity of 1 sun. Encapsulation‐free, small‐area devices operating under open circuit conditions were set up in a dry room with 1‐sun light. During LS, the device temperatures ranged from 46 to 51 °C. After exposure for 3600 s, ternary devices maintained >50% of their original PCEs (Figure S29, Supporting Information). Furthermore, optimized ternary small‐area devices showed good atmospheric stability. After measuring the ternary devices N_2_ atmospheric stability for 1000 h, it was shown to have good stability at 92% (Figure 5h; Figure S30, Supporting Information). Additionally, at 30% and 60% RH conditions, the PCEs maintained at >90% and >80% of their original PCEs (Figure S31a,b, Supporting Information), and the thermal stabilities of ternary devices were improved (Figure S31c, Supporting Information). These results demonstrate that the inclusion of BTA‐HD‐Rh resulted in remarkable solar sub‐module performance for industrial applications.

## 3 Conclusion

We designed and synthesized a new NFA (BTA‐HD‐Rh) with hexyl‐decyl side chains on a rhodanine end group. Upon mixing BTA‐HD‐Rh with PM6:L8‐BO and processed by environmentally benign non‐halogen solvents (*O*‐XY:CS_2_), the resulting ternary blend (PM6:L8‐BO:BTA‐HD‐Rh) device achieved excellent PCEs. Interestingly, we found increase in the solubility, absorption coefficient with reduced excess aggregation by varying the alkyl chain from ethyl hexyl to hexyl decyl on rhodanine group of NFA (BTA‐ERh to BTA‐HD‐Rh). The high performance of BTA‐HD‐Rh doped ternary blend over BTA‐ERh could be attributed to the improved exciton dissociation/splitting efficiency, charge carrier transport properties, as well as weak recombinations promoted higher *V*_oc_, *J*_sc_, and FF values of 9.49 V, 2.50 mA cm^−2^, 67.36%, respectively. In addition, *O*‐XY:CS_2_ processed ternary blend sub‐module (55 cm^2^) was fabricated by bar‐coating, and realized phenomenal PCEs of >16%, which is the highest PCE of sub‐modules using non‐halogen solvents. These findings demonstrate that side chain engineering is an effective strategy for creating NFA with high‐performance and environmental‐friendly solvent processability. Both attributes are critical for the commercialization of large‐area solar sub‐modules.

## 4 Experimental Section

Supporting Information contains experimental details and further characterization information.

## Conflict of Interest

The authors declare no conflict of interest.

## Supporting information

Supporting Information

## Data Availability

The data that support the findings of this study are available in the supplementary material of this article.
