# Supplementary material for: Accomplishing High‐Performance Organic Solar Sub‐Modules (≈55 cm2) with >16% Efficiency by Controlling the Aggregation of an Engineered Non‐Fullerene Acceptor
Source: Adv Sci (Weinh). 2024 Jun 18;11(31):2404997. doi: 10.1002/advs.202404997 (PMC11336910; doi:10.1002/advs.202404997)
Supplement: Supplementary file 1 — Supporting Information [file ADVS-11-2404997-s001.docx]

**Supporting Information**

**Accomplishing High-Performance Organic Solar Sub-Modules (~55 cm^2^) with >16% Efficiency by Controlling the Aggregation of an Engineered Non-Fullerene Acceptor**

*Thavamani Gokulnath, Hyerin Kim, Kakaraparthi Kranthiraja, Bo Hyeon Cho, Ho-Yeol Park, Jesung Jee, Young Yong Kim, Jinhwan Yoon** *and Sung-Ho Jin**

Dr. T. Gokulnath, H. Kim, Dr. K. Kranthiraja, B. H. Cho, Dr. H.-Y. Park, Dr. J. Jee, Prof. J. Yoon and Prof. S.-H. Jin

Department of Chemistry Education, Graduate Department of Chemical Materials, Institute for Plastic Information and Energy Materials, Sustainable Utilization of Photovoltaic Energy Research Center (ERC), Pusan National University, Busandaehakro 63-2, Busan 46241, Republic of Korea.

*E-mail addresses: jinhwan@pusan.ac.kr (J. Yoon), shjin@pusan.ac.kr (S.-H. Jin)

Dr. K. Kranthiraja

Center for Material Science, Department of Chemistry and Physics, Queensland University of Technology, Australia.

Dr. Y. Y. Kim

Beamline Division, Pohang Accelerator Laboratory, Pohang University of Science and Technology, Pohang 37673, Republic of Korea

**Experimental Procedure**

**1. Materials:** PM6 polymer and non-fullerene acceptors L8-BO, Y7-BO, Y6-BO, and BTP-eC9 were purchased from Solarmer materials, China. All supplementary materials (ITO-glass, transport layers, electrode metals, etc.) were purchased from commercial sources. Materials and supplementary materials were used without further purification. 1,3,5-trichlorobenzene was received from Sigma Aldrich, South Korea. All solvents (*o*-xylene (*O*-XY, Bp: 144 ^o^C), carbon disulfide (CS_2_, Bp: 46.24 ^o^C), chlorobenzene (CB, Bp: 131 ^o^C), and chloroform (CF, Bp: 61.2 ^o^C) used in this study were procured from Sigma Aldrich.

**2. Organic Sub-Modules Fabrication:** The pre-patterned large-area ITO-glass substrates were cleaned with acetone and isopropyl alcohol solvents. For the sub-module (area size= 55 cm^2^), the ZnO NPs (30 nm), active layer was coated onto UV ozone-treated (30 min) glass/ITO using a bar coater manufactured by printed electro-mechanical system (PEMS) (Republic of Korea).^[1,2]^ The bar speed was kept at 8 mm/sec for the deposition of the ZnO NPs (12/7 uL) layer and annealed at 110 °C for 10 min. Optimization of the solar sub-modules was carried out via multiple experimental designs, such as varying the volume of the solution to change the active layer’s thickness, and optimizing annealing conditions, etc (Table S3). All active layer blend solutions (PM6:L8-BO, PM6:BTA-HD-Rh binary (1:1.2, wt ratio) and PM6:L8-BO:BTA-HD-Rh ternary blends (1:1:0.2, 1:0.8:0.4, 1:0.6:0.6, 1:0.4:0.8, wt ratio)) with a total solids concentration of 15 mg/ml was prepared by dissolving the components in 1 ml of environmentally benign non-halogen (*O*-XY:CS_2_, v/v = 1:0, 0.2:0.8, 0.6:0.4, 0.8:0.2, and 0:1) solvents and 1,3,5-trichlorobenzene ^[3a]^ (2 mg/ml) as additive. Solutions were stirred at 70 ^o^C for 2 hours to ensure complete dissolution. Coating bar speed (15 mm/sec) and concentration of the solution (15/13 uL) were fixed for the fabrication of solar sub-modules [For thicker film fabrication for sub-modules, the variation of thickness was controlled by changing the total blend concentration and the bar speed]. During the fabrication of air-processed samples, relative humidity (RH) ranged between 30~45% and room temperature (RT) is 25 ± 3 ℃. After drying the active layer films, the sub-module substrates were heated on a hot plate at a moderate temperature of 120 °C for 20 min. Finally, 5 nm of MoO_3_ and 150 nm of Ag were thermally evaporated to complete the module in a vacuum chamber with a base pressure of 1×10^−6^ Torr.

The defined active area of sub-module is 55 cm^2^ and the geometric fill factor (GFF) in the sub-module structure was calculated by photoactive area/total area × 100 = {(0.5 cm × 10 cm) × 11 / (0.8 cm × 10 cm) × 11} × 100 = 55/88×100 = 62.5%.

**3. Small-Area Device Fabrication:** An inverted device architecture was used in this study with the device architecture: ITO-glass/ZnO/PFN-Br/active layer/MoO_3_/Ag.^[1]^ First, ITO-glass substrates were washed with detergent in deionized water, followed by ultrasonication in acetone and isopropyl alcohol solvents. After drying of the substrates, UV-ozone treatment of the substrates was carried out for 20 minutes, and a ZnO precursor solution ^[1]^ was spin-coated at 3000 rpm for 40 s and then annealed in air at 175 ^o^C for 25 minutes to obtain a ~30 nm sol-gel ZnO thin film. For electron interfacial layer, the PFN-Br was coated on ZnO at 5000 rpm for 40 s by using concentration of solution (1 mg/ml in methanol) and annealed at 70 °C for 5 min.^[4]^ All active layer blend solutions (PM6:L8-BO (1:1.2, wt ratio), PM6:L8-BO:BTA-HD-Rh (1:1:0.2, wt ratio)) with a total solids concentration of 15 mg/ml was prepared by dissolving the components in 1 ml of environmentally benign non-halogen solvents (*O*-XY:CS_2_, v/v = 0.8:0.2) and 1,3,5-trichlorobenzene as additive (stirring at 70 ^o^C for 2 hours, the solution for several hours to ensure complete dissolution). An active solution was spin-coated in air atmosphere onto the ITO/ZnO/PFN-Br substrates using the same coating speed. During the fabrication of all samples, ambient humidity ranged between 30~45% (RH) and RT is 24 ± 3 ℃. Then all samples treated with thermal annealing at 100 °C for 10 min. Finally, small-area devices were completed by thermal evaporation of 6 nm of MoO_3_ and 150 nm of Ag. Small-area device had an active contact area of 0.04 cm^2^. The efficiency of devices was tested in a dry room condition without encapsulation.

**4. Current-Voltage Analysis:** Power conversion efficiency (PCE) was measured under AM (air mass) 1.5G illumination at 100 mW cm^-2^ using a AAA solar simulator (Oriel Sol3A Class AAA solar simulator, model 940443A) calibrated with a standard photovoltaic cell equipped with a KG5 filter and computer controlled Keithley 2400 source measure unit. All sub-module (area is 55 cm^2^) device PCEs were measured in dry room condition.

**5. J_ph_-V_eff_ Measurement:** Here, *J*_ph_ is like J_L_-J_D_, where J_L_ and J_D_ are the current density in the light and dark, respectively. *V*_eff_ is the difference between *V*_0_ and *V*_appl_, where *V*_appl_ is the applied bias voltage and *V*_0_ is the voltage when *J*_ph_ = 0.

**6. EQE Analysis:** The incident photon to current conversion efficiency spectrum was measured using Oriel^®^ IQE-200^TM^ equipped with a 250 W quartz tungsten halogen lamp as the light source and a monochromator, an optical chopper, a lock-in amplifier, and a calibrated silicon photodetector. Prior to the use of the light, the spectral response and the light intensity were calibrated using a monosilicon detector.

**7. Trap-Assisted Recombination:** The slope of the semi-log plot of *V_oc_* versus P_light_ was determined using the formula; *V*oc ∝ n(*k*T/q)ln(P_light_), (where *k* is the Boltzmann constant, T is the temperature, and q is the elementary charge), and this slope can be used to investigate trap-assisted recombination.

**8. Charge Carrier Mobility Measurements:** Hole-only and electron-only devices were fabricated to measure the hole and electron mobilities of active layers using the space charge limited current (SCLC) method with hole-only device of ITO/PEDOT:PSS/active layer/MoO_3_/Ag and electron-only device of ITO/ZnO NPs/active layer/PDINO/Ag. The SCLC mobilities were calculated by MOTT-Gurney equation, which is described by:  *J*= 9/8 ε_0_ε_r_*μV*^2^/*d*^3^,

where *J* is the current, *E* is the effective electric field, ε_0_ is the permittivity of free space (8.85×10^-12^ F m^-1^), ε_r_ is the material relative permittivity, *d* is the thickness of the active layer, *μ* is the hole or electron mobility and *V* is the effective voltage. The effective voltage can be obtained by subtracting the built-in voltage (*V*_bi_) and the voltage drop (*V*_s_) from the substrate’s series resistance from the applied voltage (*V*_appl_), *V* = *V*_appl_– *V*_bi_ – *V*_s_. The mobility can be calculated from the slope of the *J*^1/2^ ~V curves.

**9. Trap-State Density Measurement:** The trap-state density was determined according to the formula; *N*_t_ = (2*ε_r_ε*_0_*V*_TFL_)/(qL^2^),

where, *N*_t_ is the trap-state density, *V*_TFL_ is the trap-filling limit voltage, ε_r_ is the relative dielectric constant (3), ε_0_ is the vacuum permittivity (8.85×10^-14^ CV^-1^ cm^-1^), L is the thickness of active layer (⁓140 nm), and q is the elementary charge (1.6×10^-19^ C).

**10. UV-Vis Absorbance:** The absorption spectra of the pristine and blend states were measured on a UV-1800 spectrophotometer (Shimadzu Scientific Instruments) and Hitachi F-4500 fluorescence spectrophotometers at room temperature.

The absorption coefficient (α) value was calculated using the following equation; (α) = 2.303*(A/l) (in cm), where A-absorbance and l-thickness (*Energy Environ. Sci.,* **2017**, *10,* 258)

**11. Active Layer Thickness Analysis:** The thickness of active layers was measured via the KLA Tencor Alpha-step IQ surface profilometer.

**12. Morphological Analysis:** The surface morphology of the blend films was characterized by AFM images were acquired with a XE-100 (park system corp) in tapping mode. Samples were prepared under optimized conditions and the measurement was carried out in tapping mode. For bulk morphology analysis, TEM images were acquired with a H-7600 (HITACHI)/electron microscopy.

**13. Contact Angles Study:** The contact angles were measured using a contact angle 101 measuring system (Plasma systems and materials, South Korea).

**14. Flory–Huggins Interaction Parameter:** The Flory–Huggins interaction parameter was also calculated using the following equation with the surface energy values of the donor and acceptor component (*γ*_donor_ and *γ*_acceptor_, respectively): χ∝[(*γ*_donor_)^2^-(*γ*_acceptor_)^2^]^2^, χ = Flory–Huggins interaction parameter and *γ* = Surface energy.

**15. PL Measurement:** Photoluminescence (PL) measurements were carried out using compact fluorescence lifetime spectrometer C11367 at room temperature.

**16. Impedance Study:** The impedance response was measured over the range of 1 Hz to 1 MHz with oscillation amplitude of 15 mV under dark condition (Bio-Logic VMP-3). The experimental data were simulated using commercial Z-view software to estimate the values for each component of the corresponding equivalent circuits.

**17. GIXRD Characterizations:**  GIWAXS measurements were carried out using a synchrotron X-ray radiation source (λ = 0.1274 nm) at the 3C beamline of the Pohang Accelerator Laboratory, in Pohang, South Korea. Samples were prepared on glass substrates, and the incident angle of the X-ray beam was set to an intermediate value between the critical angles of the films and the glass substrate. GIWAXS signals were collected by a two-dimensional Eiger4M single photon count detector (Dectris, switzerland) at the sample-to-detector distance of 221 mm. The obtained data were analyzed with p-GIXS.^[3b]^

**18. Device Stability Analysis:** Under simulated one sun continuous illumination for up to 1 hour, small-area devices were tested (photo stability) in a dry room environment condition. The devices' temperatures under light soaking ranged between 25 to 30 °C. An encapsulated small-area and sub-module (covered with aluminium foil) devices were tested for stability in both the open air and the nitrogen atmosphere. Moreover, thermal stability was measured in a dry environment at 80 °C while being continuously heated.

**Synthesis Procedure of New Non-Fullerene Acceptor (BTA-HD-Rh).**

**
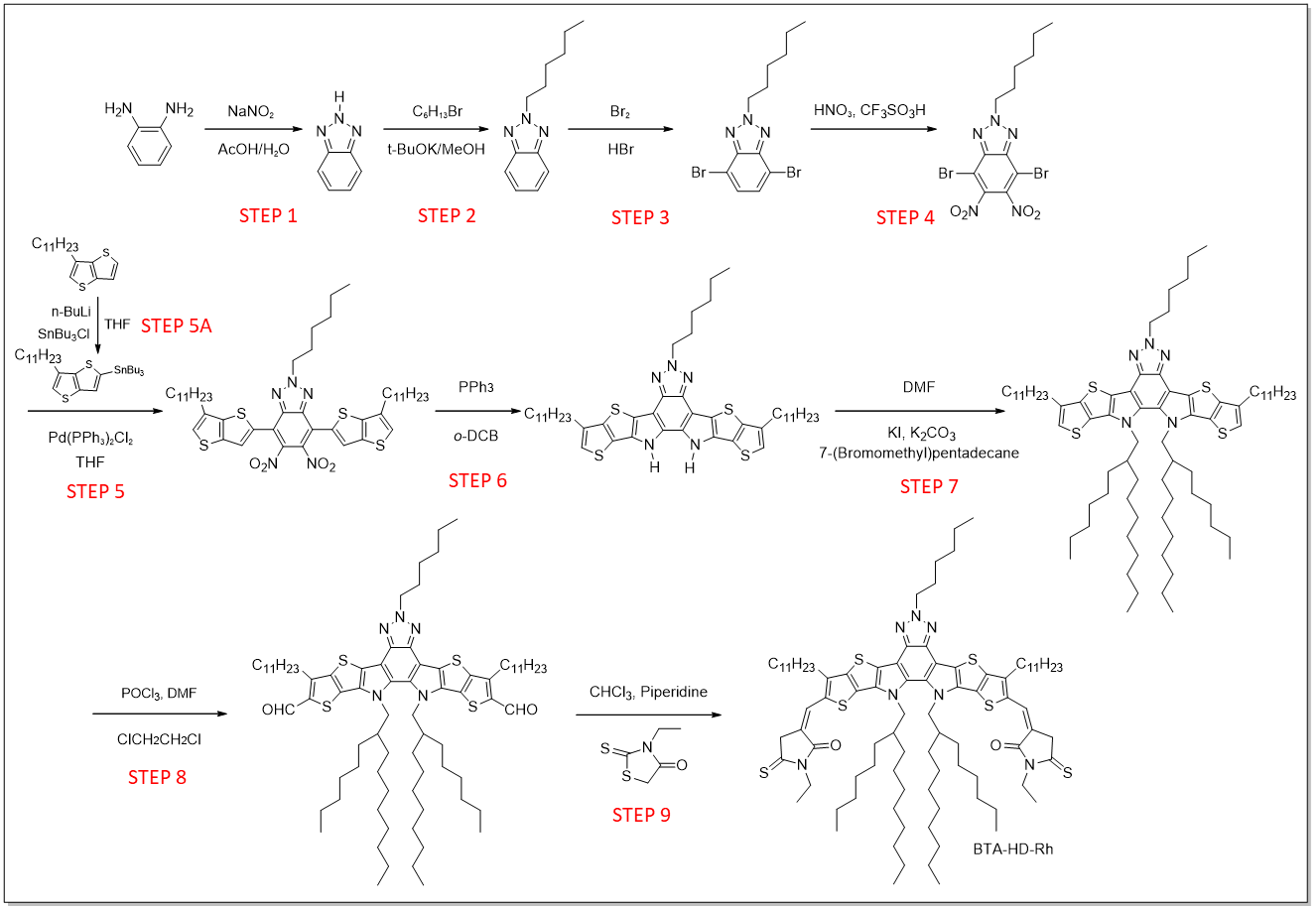
**

**Scheme S1.** Synthetic route of new non-fullerene acceptor (BTA-HD-Rh).

**Synthesis of 2H-benzo[d][1,2,3]triazole (1)**

Benzene-1,2-diamine (10g, 92.47mmol) and aqueous solution of NaNO_2_ (7.02g, 101.72mmol) was dissolved in CH_3_COOH (150mL). After stirring at room temperature for 1hr, the mixture was extracted with ethyl acetate. Combined organic layer was concentrated under vacuum and dried over Na_2_SO_4_. The crude product was purified by silica gel column chromatography using hexane/ethyl Acetate (1:1, v/v). Compound **1** was obtained as white solid (10.3g, 93% yield). ^1^H NMR (400 MHz, CDCl_3_): δ (ppm) 7.98-7.96(m, 2H), 7.50-7.48(m, 2H).

**Synthesis of 2-hexyl-2H-benzo[d][1,2,3]triazole (2)**

Compound **1** (5g, 41.97mmol) and potassium tert-butoxide (4.95g, 44.07mmol) was dissolved in methanol (25mL) and 1-Bromohexane (6.87mL, 48.69mmol) was added dropwise under nitrogen atmosphere. The reaction mixture was stirred at 65℃ for 24hr. The mixture was extracted with chloroform and combined organic layer was dried over Na_2_SO_4_. The product was purified with column chromatography on silica gel using hexane/ethyl acetate (4:1, v/v). Compound **2** obtained as colorless oil (4.4g, 51% yield). ^1^H NMR (400 MHz, CDCl_3_): δ (ppm) 7.80-7.78 (m, 2H), 7.31-7.29 (m, 2H), 4.67-4.63 (t, 2H), 2.06-2.03 (m, 2H), 1.29-1.22 (m, 6H), 0.82-0.87 (t, 3H).

**Synthesis of 4,7-dibromo-2-hexyl-2H-benzo[d][1,2,3]triazole (3)**

In a 500mL round-neck flask, Compound **2** (4.13g, 20.31mmol) and HBr solution (24.4mL) were added and stirred at 100℃ for 1hr. Bromine (3.6mL, 63.36mmol) was added dropwise. The reaction was heated to 135℃ overnight. After that mixture was cooled to room temperature. After cooling to RT, Aqueous solution of sodium thiosulfate was added and the aqueous layer was extracted with dichloromethane and dried over anhydrous MgSO_4_. The crude product was purified by silica gel column chromatography using hexane/dichloromethane (7:3, v/v). Light yellow oil was obtained (3.1g, 44% yield). ^1^H NMR (400 MHz, CDCl_3_): δ (ppm) 7.46 (s, 2H), 4.82-4.78(t, 2H), 2.20-2.13 (m, 2H), 1.41-1.33 (m, 6H), 0.91-0.88 (t, 3H).

**Synthesis of 4,7-dibromo-2-hexyl-5,6-dinitro-2H-benzo[d][1,2,3]triazole (4)**

Nitric acid (5.651g, 83.08mmol) was added dropwise to trifluoromethane sulfonic acid (13.2mL, 124.6mmol) at 0℃. After stirring for few minutes, compound **3** (3g, 8.30mmol) was added at same temperature. The reaction mixture was stirred at 80℃ for 3hr. After cooling to RT, the reaction mixture was poured into ice water and the aqueous layer was extracted with ethyl acetate. The organic layer was dried over MgSO_4_ and the solvents were evaporated under reduced pressure. The crude product was purified by column chromatography on silica gel using hexane/ethyl acetate (4:1, v/v). Yellow solid was obtained (3.4g, 64% yield). ^1^H NMR (400 MHz, CDCl_3_): δ (ppm) 4.90-4.86 (t, 2H), 2.24-2.17 (m, 2H), 1.42-1.28 (m, 6H), 0.94-0.88 (t, 3H).

**Synthesis of tributyl(6-undecylthieno[3,2-b]thiophen-2-yl)stannane (5)**

In a 100mL two-neck round-bottom flask thieno[3,2-b]thiophene (2.50g, 8.49mmol) was dissolved in THF (50mL) under the protection of nitrogen. 2.5M n-butyllithium (3.7mL, 9.3mmol) dropwise at -78℃. After stirring at -78℃ for 1hr, tributyltin chloride (2.53mL, 9.33mmol) was added in one portion to the mixture at same temperature. Mixture was gradually warmed up to room temperature and stirred for overnight. After stirring, the reaction mixture was quenched with water and extracted with dichloromethane. The organic layer was dried over Na_2_SO_4_ and removing the solvents under reduced pressure gave a tan oil. Without any further purification process, the product was used in the next step reaction.

**Synthesis of 2-hexyl-5,6-dinitro-4,7-bis(6-undecylthieno[3,2-b]thiophene-2-yl)-2H-benzo[d][1,2,3]triazole (6)**

Compound **4** (2.5g, 5.54mmol) and compound **5a** (12.9g, 22.16mmol) were dissolved in THF (24mL) and then Pd(PPh_3_)_2_Cl_2_ (0.39g, 0.55mmol) was added as catalyst. The reaction mixture was stirred for 16hr under nitrogen at 70℃. After the reaction, water was added to quench the reaction, and the mixture was extracted with dichloromethane and organic layer was dried over Na_2_SO_4_. The product was purified by column chromatography on silica gel using hexane/dichloromethane (7:3, v/v) as an eluent. Red solid was obtained (2.95g, 60% yield). ^1^H NMR (400 MHz, CDCl_3_): δ (ppm) 7.67 (s, 2H), 7.07 (s, 2H), 4.79-4.75 (t, 2H), 2.72-2.68 (t, 4H), 2.10-2.07 (m, 2H), 1.73-1.67 (m, 4H), 1.30-1.19 (m, 3H), 0.83-0.80 (m, 9H).

**Synthesis of 6-hexyl-3,9-diundecyl-12,13-dihydro-6H-thieno[2'',3'':4',5']thieno[2',3':4,5]pyrrolo[3,2-g]thieno[2',3':4,5]thieno[3,2-b][1,2,3]triazolo[4,5-e]indole (7)**

In a microwave tube Compound **5** (0.5g, 0.57mmol) is dissolved with 1,2-dichlorobenzene (5mL). After that PPh_3_ (1.5g, 5.7mmol) were added and heated at 180℃ for 1hr 5min in the microwave reactor. The aqueous phase was extracted with dichloromethane and organic layer was dried over Na_2_SO_4_ and solvent was evaporated under reduced pressure. Compound **5** was obtained as a dark green liquid without further purification.

**Synthesis of 6-hexyl-12,13-bis(2-hexyldecyl)-3,9-diundecyl-12,13-dihydro-6H-thieno[2'',3'':4',5']thieno[2',3':4,5]pyrrolo[3,2-g]thieno[2',3':4,5]thieno[3,2-b][1,2,3]triazolo[4,5-e]indole (8)**

The synthesis and purification procedure are the same with compound **8a**. 2-ethylhexyl bromide is replaced with 2-hexyldecylbromide. The product was obtained as a dark orange solid (1.28 g, Yield: 41%). ^1^H NMR (400MHz, CDCl_3_): δ (ppm) 6.90 (s, 2H), 4.76-4.72 (t, 2H), 4.50-4.49 (d, 4H), 2.75-2.72 (t, 4H), 2.16-2.10 (m, 2H), 1.94-1.91 (m, 2H), 1.80-1.76 (m, 6H), 1.20-1.19 (m, 45H), 0.81-0.75 (m, 48H), 0.62-0.59 (m, 12H).

**Synthesis of 6-hexyl-12,13-bis(2-hexyldecyl)-3,9-diundecyl-12,13-dihydro-6H-thieno[2'',3'':4',5’] thieno[2',3':4,5]pyrrolo[3,2-g]thieno[2',3':4,5]thieno[3,2-b][1,2,3]triazolo[4,5-e]indole-2,10-dicarbaldehyde (9)**

The synthesis and purification procedure are the same with compound **9a**. The product was obtained as orange liquid (1.2g, 89% yield). ^1^H NMR (400 MHz, CDCl_3_): δ (ppm) 10.15 (s, 2H), 4.86-4.72 (t, 2H), 4.63-4.61 (d, 4H), 3.23-3.19 (t, 4H), 2.26-2.22 (m, 4H), 1.98-1.93 (m, 6H), 1.50-1.46 (m, 8H), 1.40-1.29 (m, 37H), 1.01-0.82 (m, 48H), 0.72-0.68 (m, 12H).

**Synthesis of (5E,5'E)-5,5'-((6-hexyl-12,13-bis(2-hexyldecyl)-3,9-diundecyl-12,13-dihydro-6H-thieno[2'',3'':4',5']thieno[2',3':4,5]pyrrolo[3,2-g]thieno[2',3':4,5]thieno[3,2-b][1,2,3]triazolo[4,5-e]indole-2,10-diyl)bis(methanylylidene))bis(3-ethyl-2-thioxothiazolidin-4-one) (BTA-HD-Rh)**

The product **BTA-HD-Rh** was obtained as a dark blue solid (250mg, 63% yield). ^1^H NMR (400 MHz, CDCl_3_): δ (ppm) 8.03 (s, 2H), 4.75-4.72 (t, 2H), 4.58-4.56 (d, 4H), 4.18-4.16 (q, 4H), 2.96 (t, 4H), 2.14-2.13 (m, 2H), 1.90-1.78 (m, 6H), 1.39 (m, 8H), 1.30-1.19 (m, 35H), 0.97-0.79 (m, 48H), 0.74-0.70 (m, 10H), 0.62-0.60 (m, 12H). ^13^C NMR (400 MHz, CDCl_3_): δ (ppm) 191.69, 167.46, 143.76, 143.34, 137.03, 135.92, 132.29, 132.00, 128.33, 125.31, 124.23, 118.17, 110.53, 39.95, 38.98, 31.92, 29.66, 29.62, 29.56, 29.42, 29.35, 22.70, 22.64, 22.53, 14.14, 14.03, 12.33. HRMS (FAB^+^): m/z (100%): calcd for C_90_H_137_N_7_O_2_S_8_; 1603.8599, Found: 1603.8605.

**
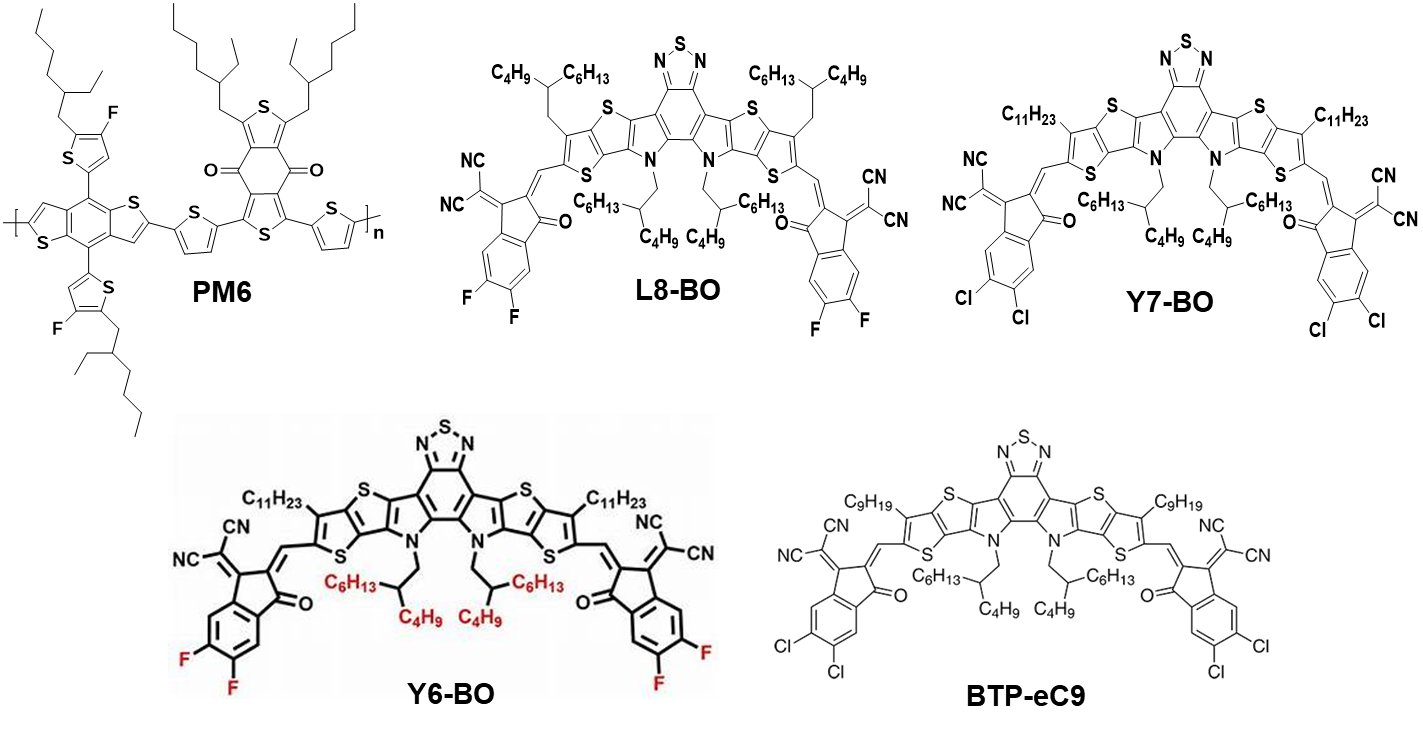
Figure S1.** Molecular structure of PM6 polymer donor and NFAs (L8-BO, Y7-BO, Y6-BO, and BTP-eC9).

**
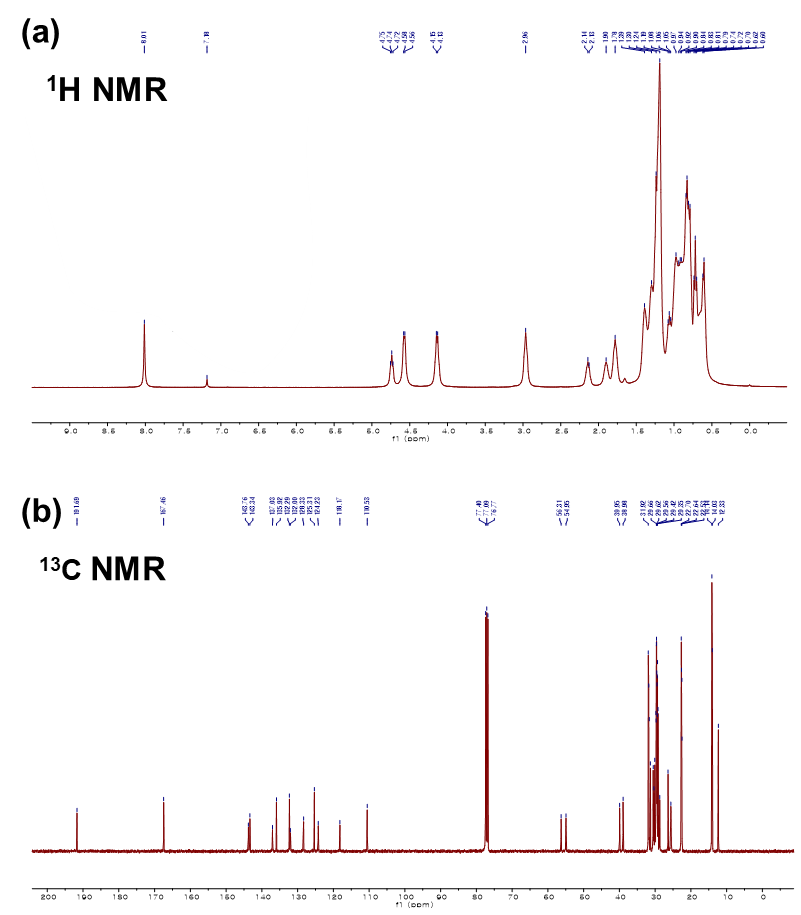
**

**Figure S2a.** (a) ^1^H NMR and (b) ^13^C NMR spectra of BTA-HD-Rh.

**Figure S2b.** Mass spectra of BTA-HD-Rh.

**
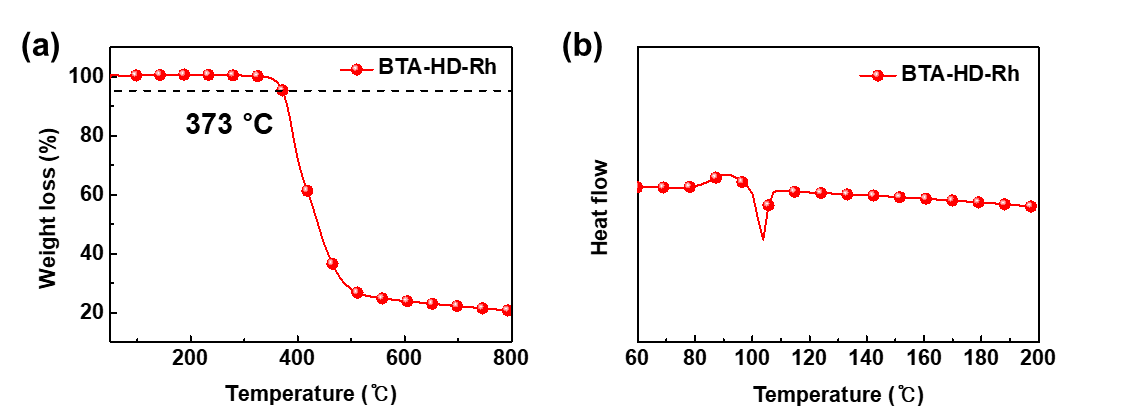
**

**Figure S3.** (a) Thermogravimetric analysis (TGA), (b) differential scanning calorimetry (DSC) curves of BTA-HD-Rh.

**
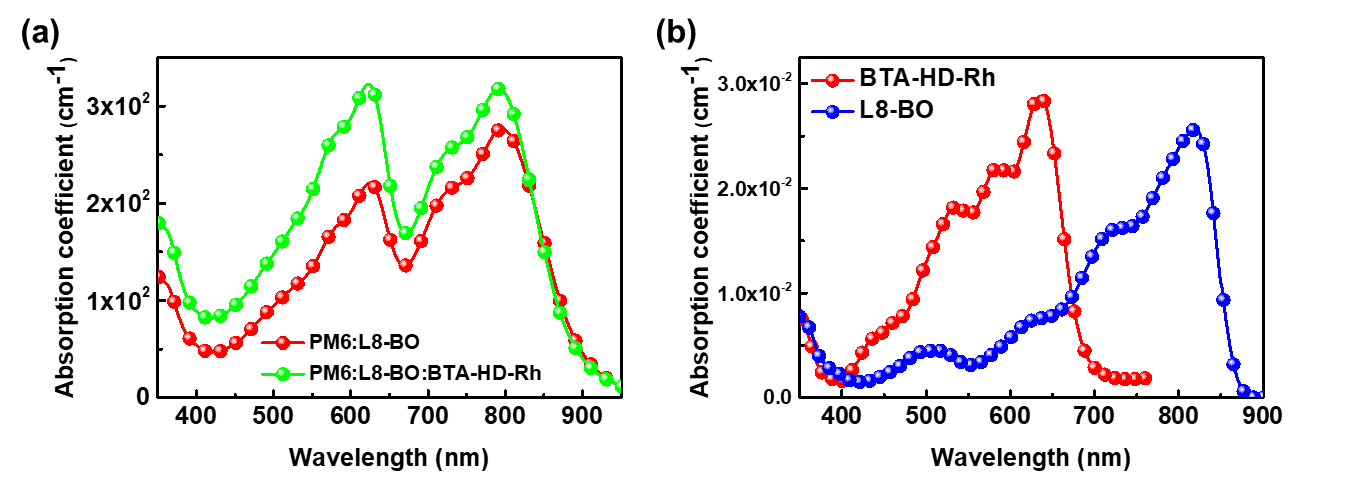
**

**Figure S4.** (a) Absorption co-efficient of optimized binary and ternary blend films. (b) Absorption co-efficient of BTA-HD-Rh and L8-BO.

**
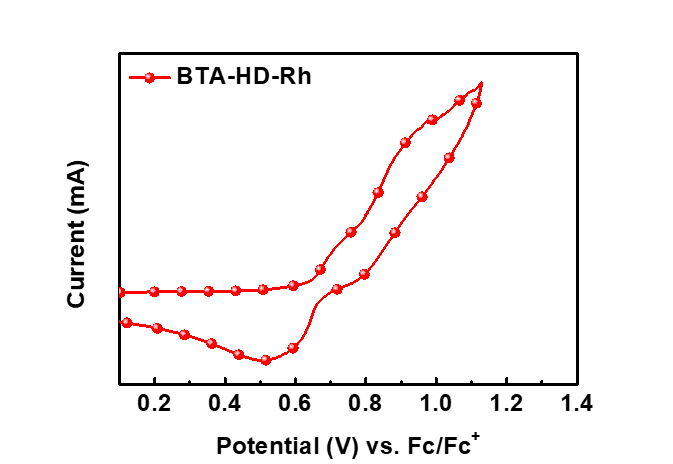
**

**Figure S5.** Cyclic voltammetry measurements for BTA-HD-Rh.

**
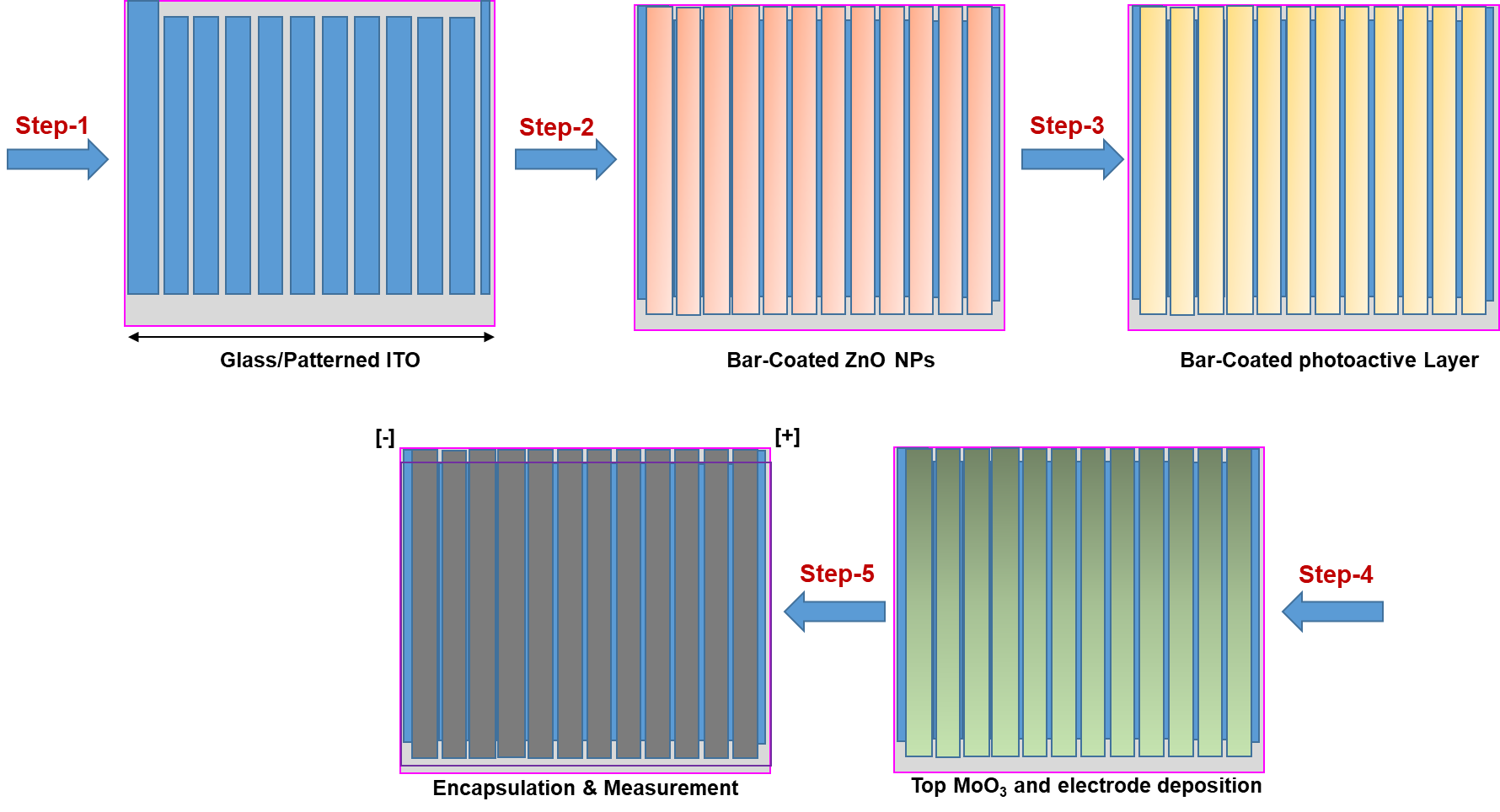
Figure S6.** Schematic diagram for preparing sub-module system using the bar coating in air-atmosphere.


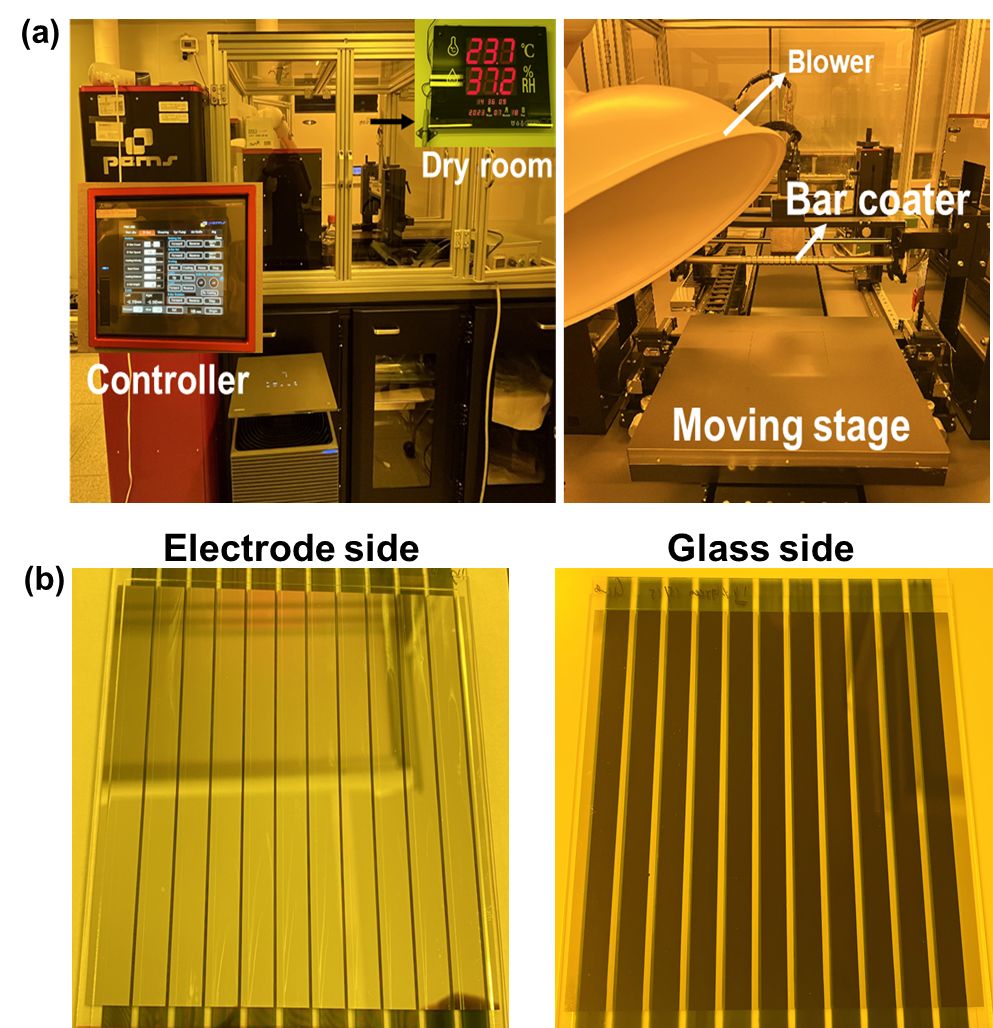


**Figure S7.** (a) The photograph pictures of bar coater equipment (produce by PEMS, Republic of Korea). (b) Images of sub-module (electrode and glass sides).


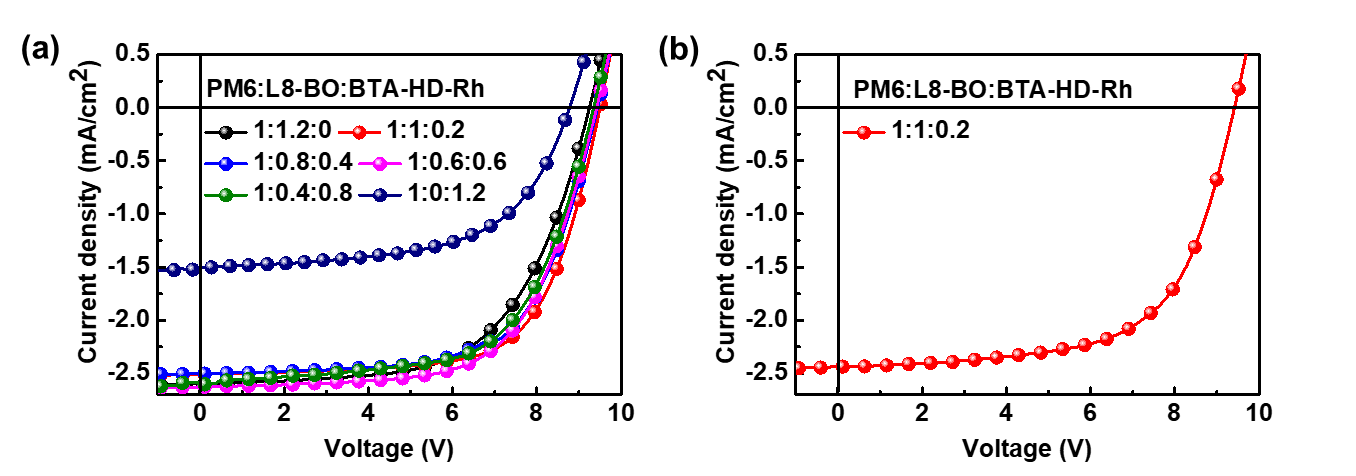


**Figure S8.** (a) Optimized *J-V* curves of binary and ternary blend sub-module devices (*O*-XY:CS_2_, v/v = 0.6:0.4). (b) Optimized *J-V* curves ternary blend sub-module with halogenated solvents (CB:CF, v/v = 0.4:0.6).

**
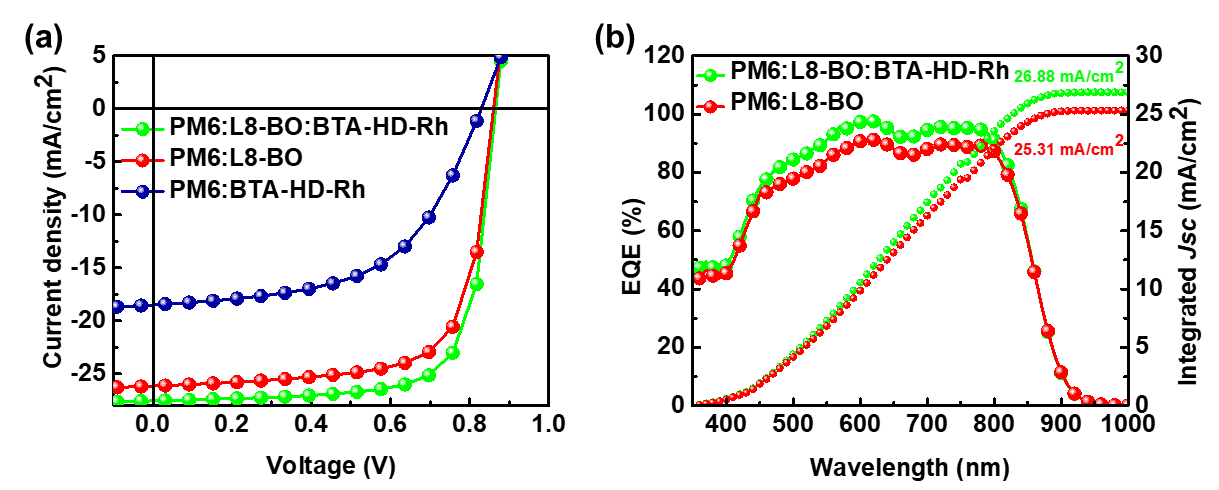
Figure S9.** (a) *J-V* curves of optimized small-area devices. (b) EQE curves of optimized small-area devices.

**
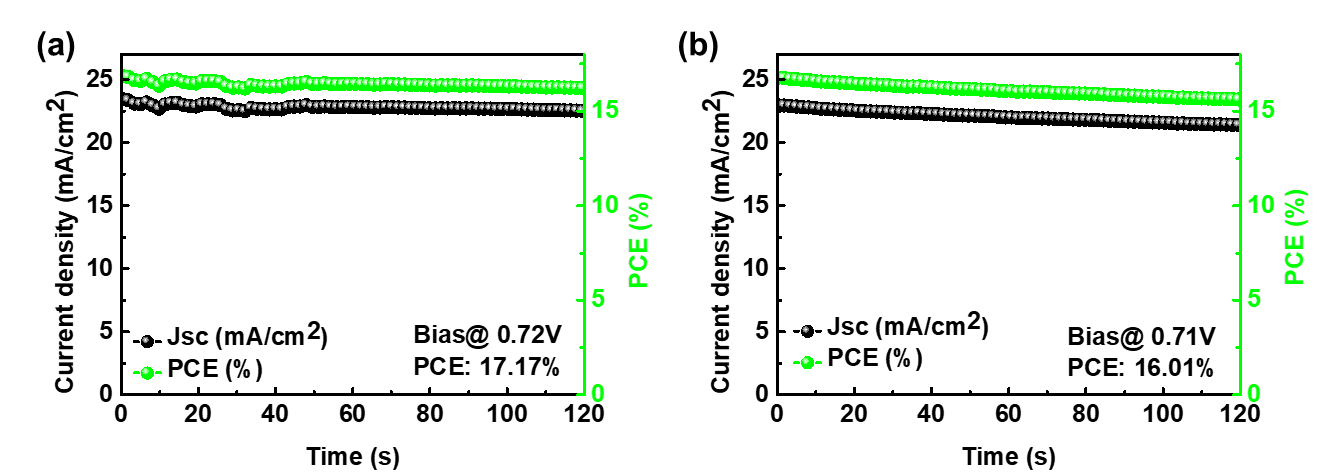
 Figure S10.** Steady-state measurement of optimized (a) PM6:L8-BO:BTA-HD-Rh, (b) PM6:L8-BO devices.

**
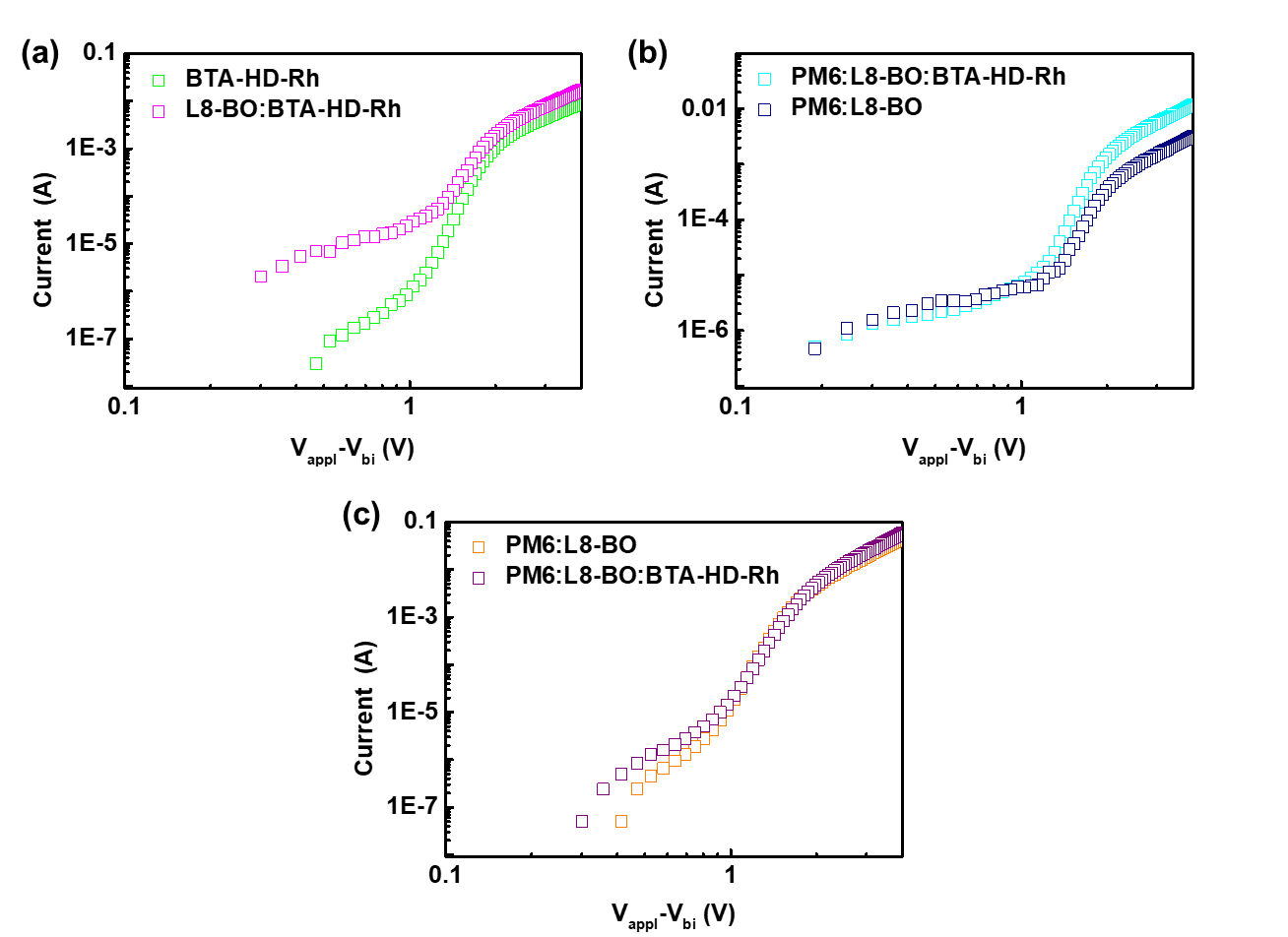
Figure S11.** SCLC (a) Electron-only of pristine and blend NFAs. (b and c) Hole and electron only of optimized binary and ternary blend film devices.


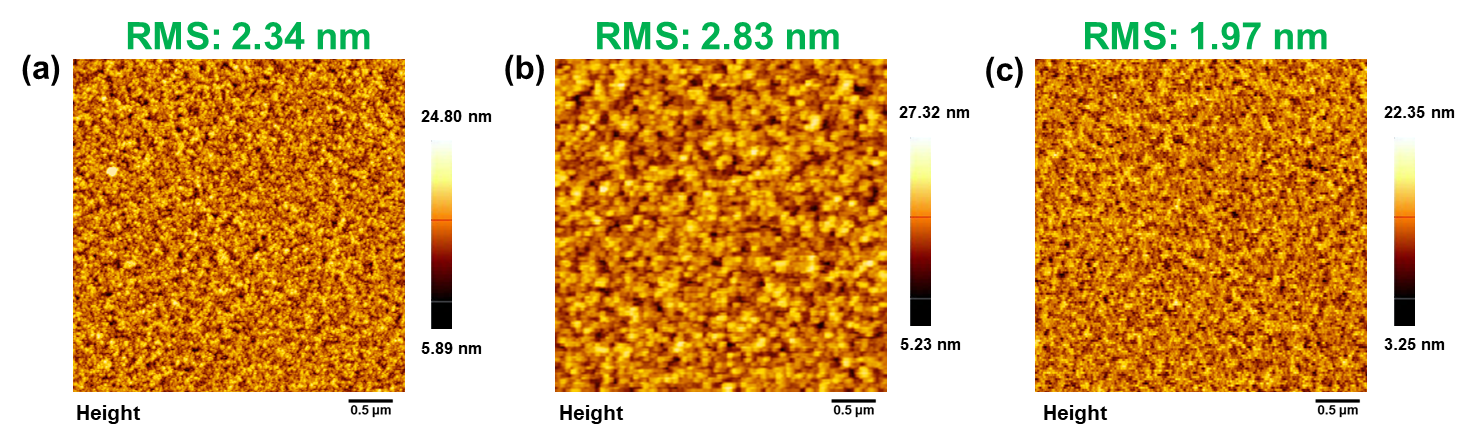


**Figure S12.** AFM images of pristine and blend NFAs, (a) BTA-HD-Rh, (b) L8-BO and (c) L8-BO:BTA-HD-Rh.

**
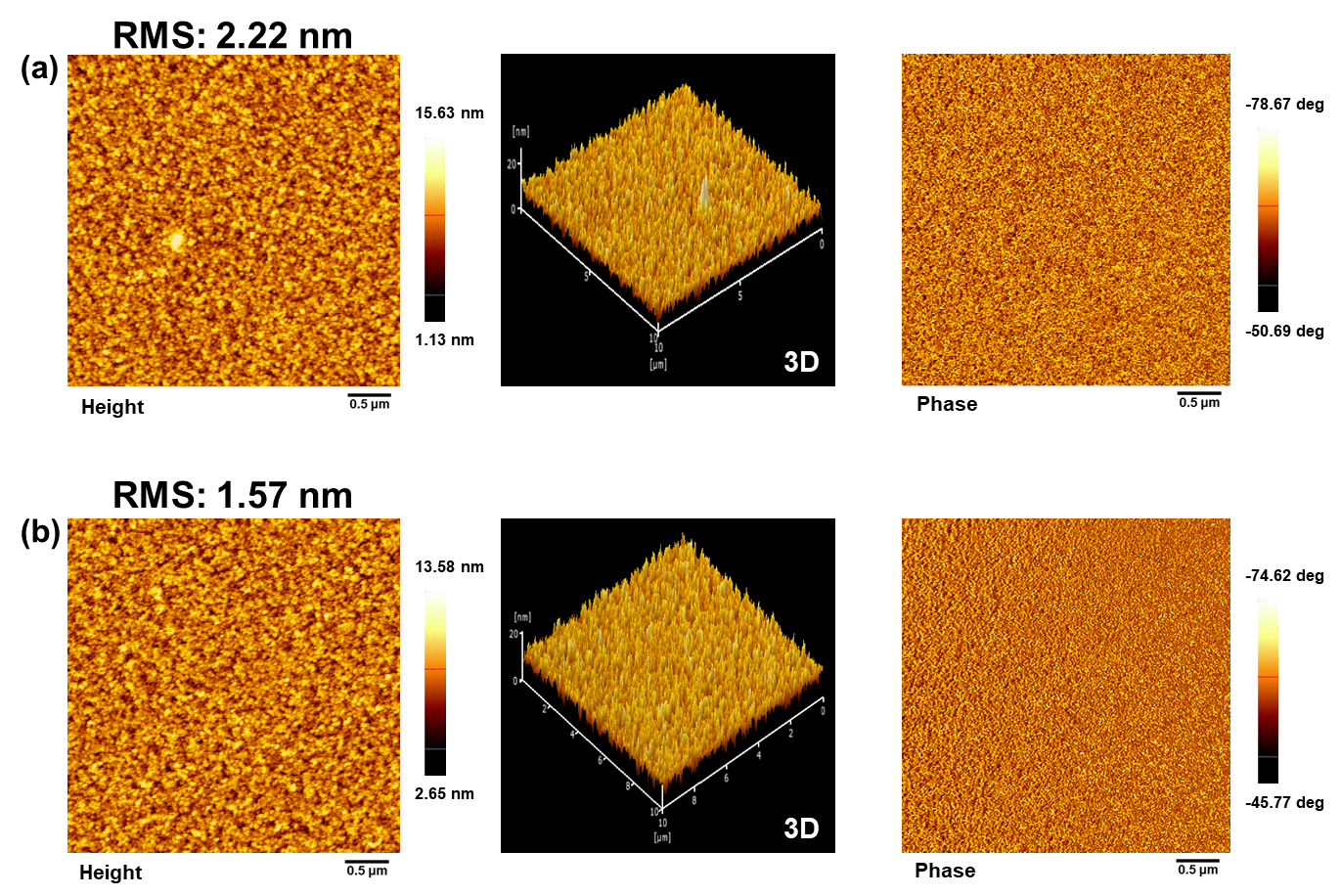
Figure S13.** AFM height, 3D, and phase images of optimized sub-module (a) PM6:L8-BO, (b) PM6:L8-BO:BTA-HD-Rh blend films.


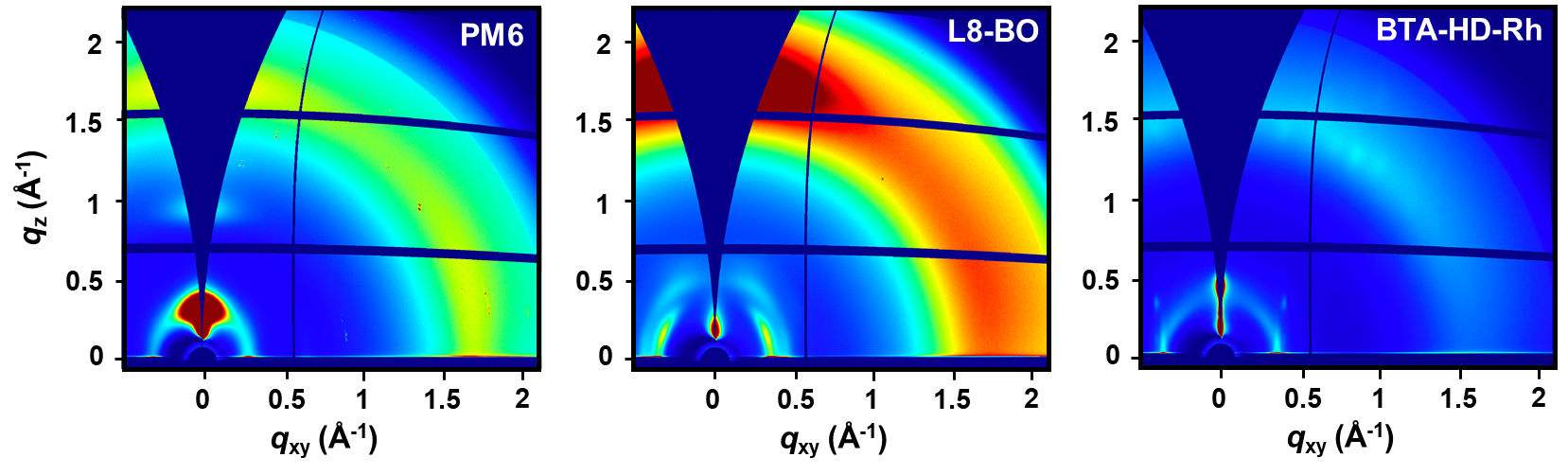


**Figure S14.** 2D-GIWAXS images of PM6, L8-BO and BTA-HD-Rh.

**
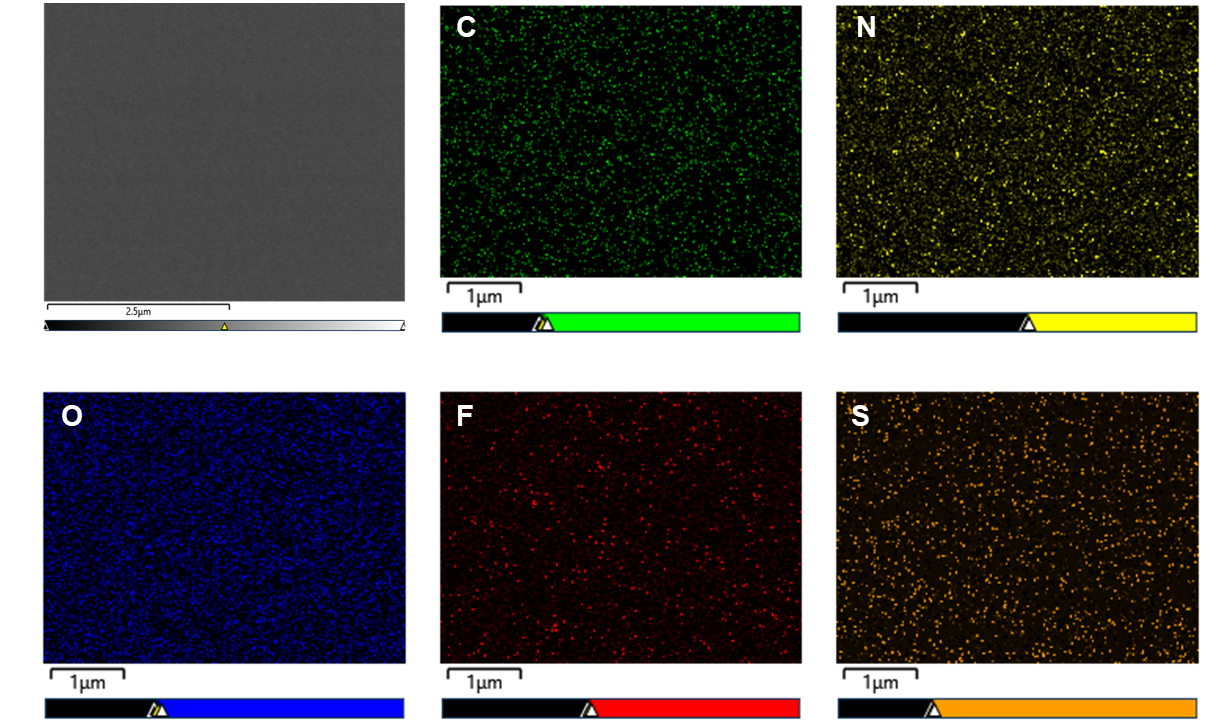
**

**Figure S15.** FE-SEM and EDS images of ternary blend film.

**
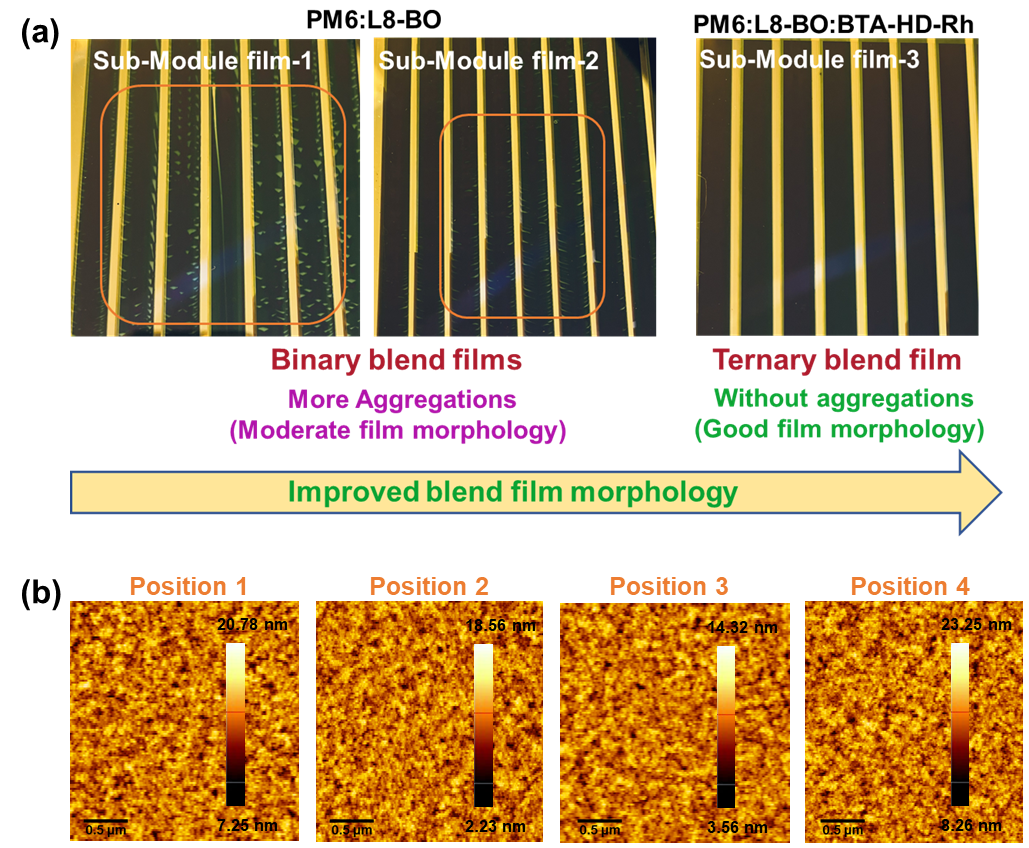
 Figure S16.** (a) Sub-module images of optimized binary and ternary blend films processed with *O*-XY:CS_2_ (v/v = 0.6:0.4) solvents. (sub-module film-1, 12 mg/ml; sub-module film-2, 15 mg/ml; and sub-module film-3, 15 mg/ml); (b) The morphology of ternary blend films at different positions.

**
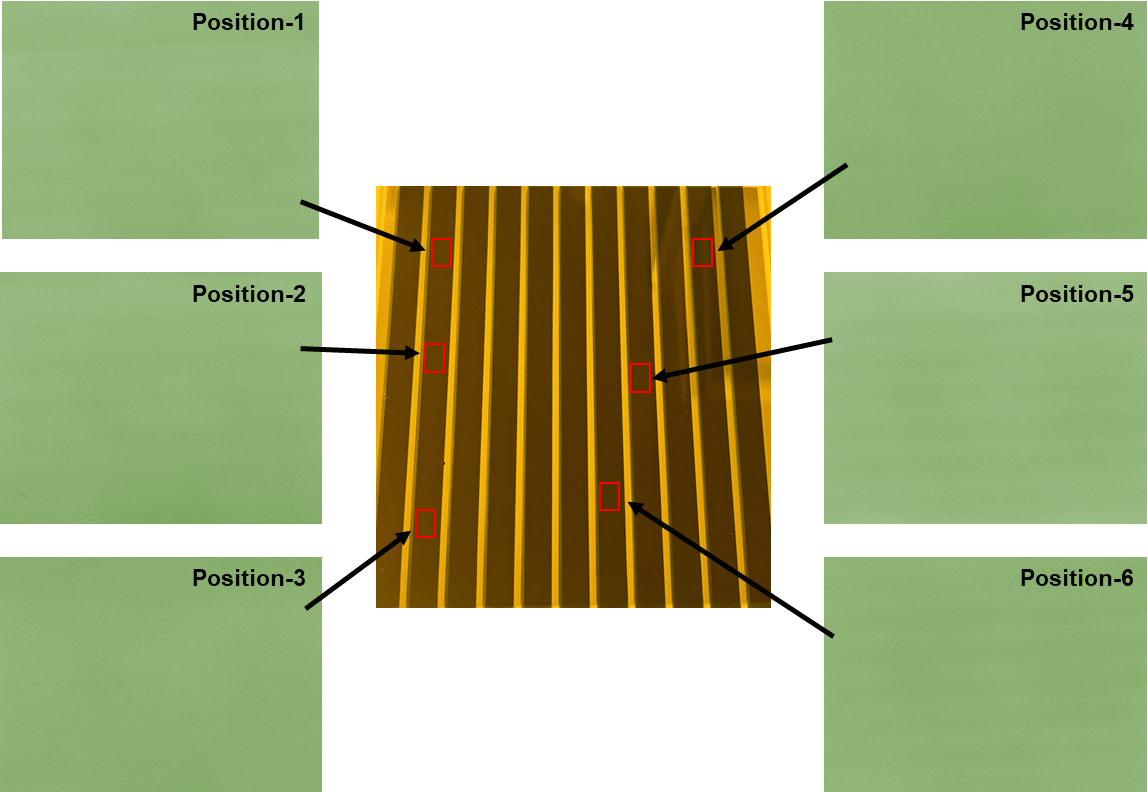
Figure S17.** Optical microscope analysis (20 μm) of ternary blend sub-module film at different positions.

**
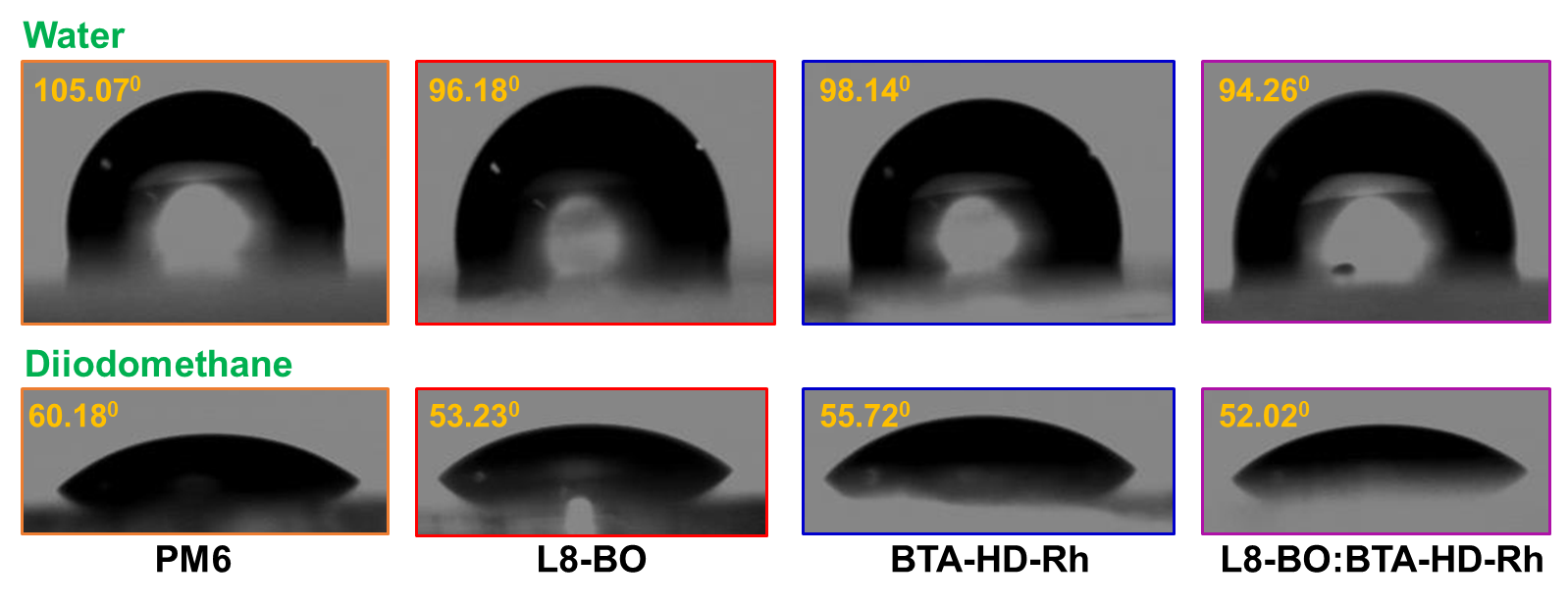
 Figure S18.** Contact angle images of optimized pristine and blend films.

**
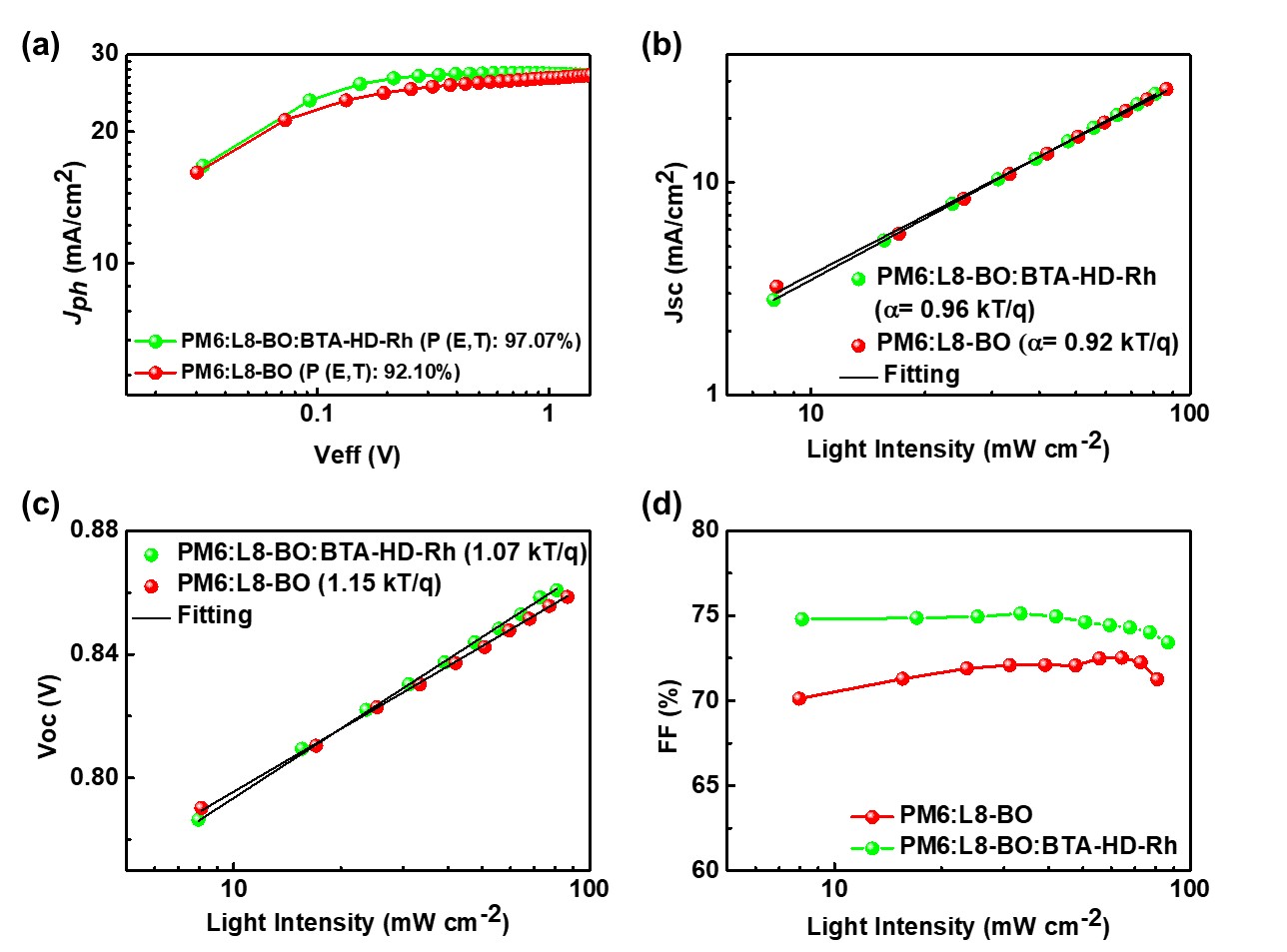
**

**Figure S19.** (a) *J_ph_* vs. *V_eff_* plots, (b) *J_sc_* vs. *P_light_* plots, (c) *V_oc_* vs. *P_light_* plots, and (d) FF vs. *P_light_* plots of optimized devices.

**
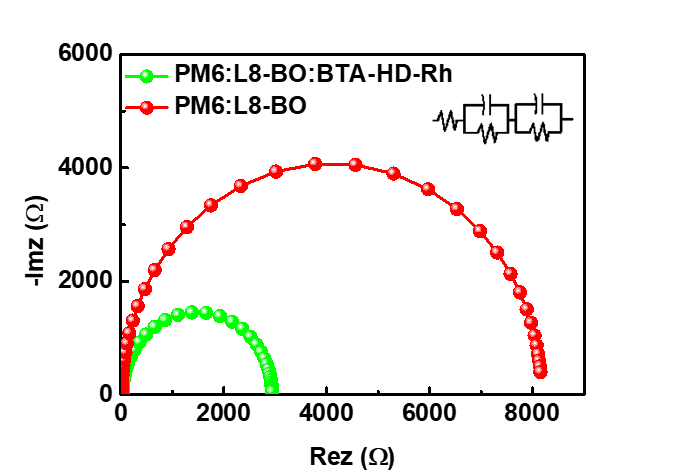
**

**Figure S20.** EIS spectra of optimized binary and ternary devices.

**
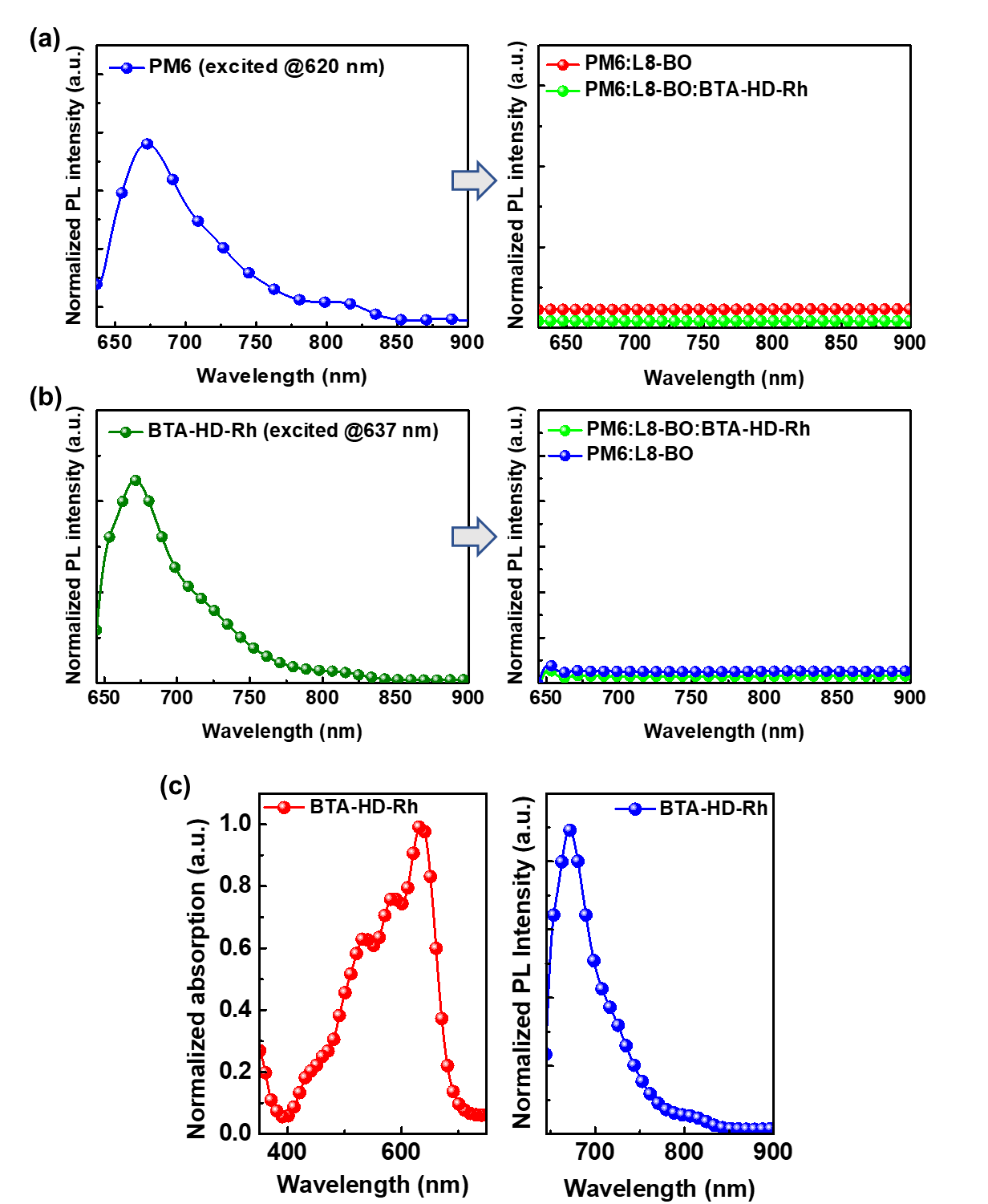
**

**Figure S21.** PL quenched spectra of optimized blend films (excited at (a) 620 nm and (b) 637 nm). (c) UV-Vis and PL spectra of BTA-HD-Rh.

**
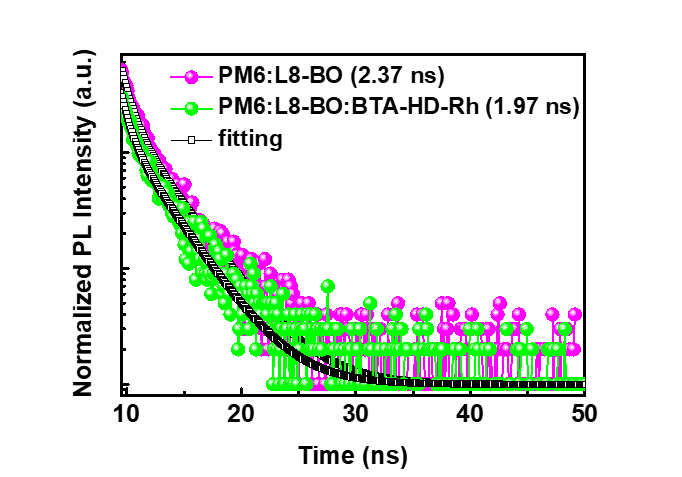
**

**Figure S22.** TRPL spectra of optimized blend films.

**
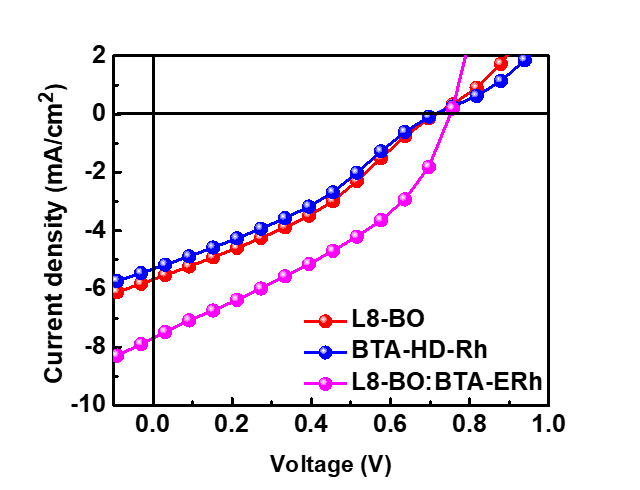
**

**Figure S23.** Optimized *J–V* curves of L8-BO, BTA-HD-Rh, L8-BO:BTA-HD-Rh based an active layers.

**
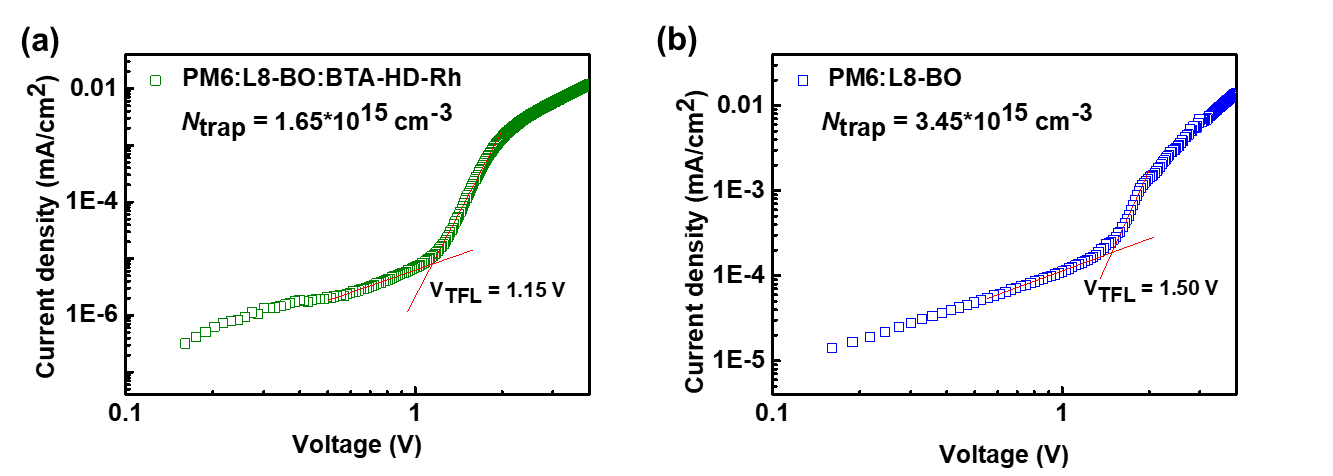
**

**Figure S24.** Trap-state densities of optimized (a) ternary and (b) binary device.


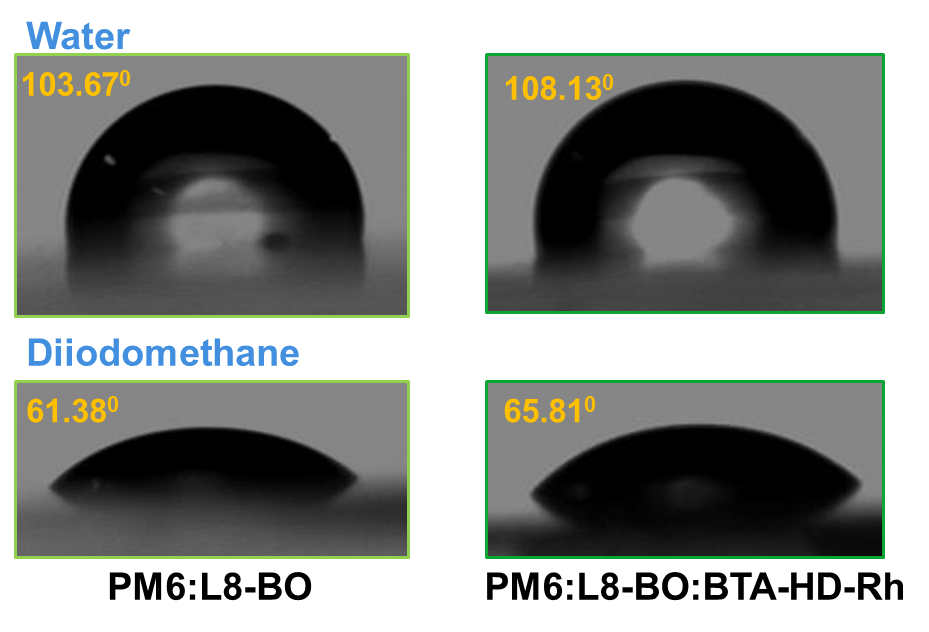


**Figure S25.** Water contact angle images of optimized binary and ternary blend films.

**
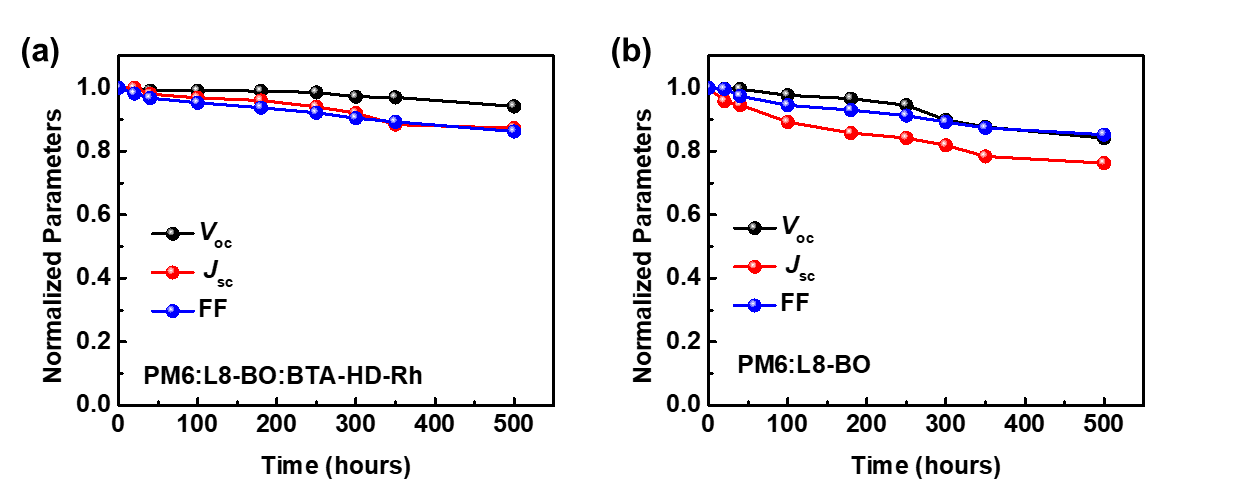
Figure S26.** Stabilities of sub-module optimized devices with low RH (25 °C, RH: 30%).

**
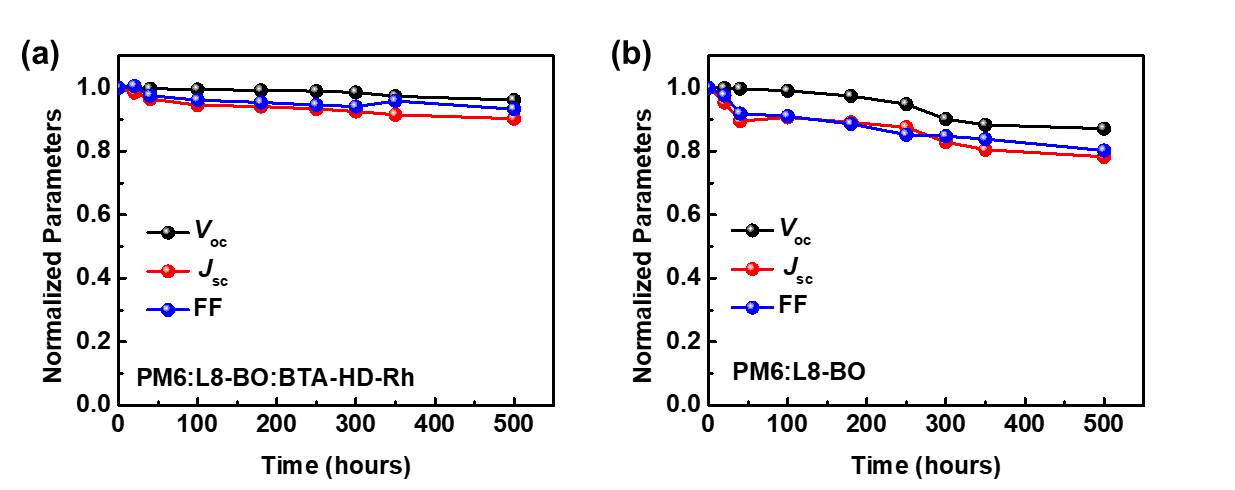
Figure S27.** Stabilities of sub-module optimized devices with high RH (22 °C, RH: 60%).

**
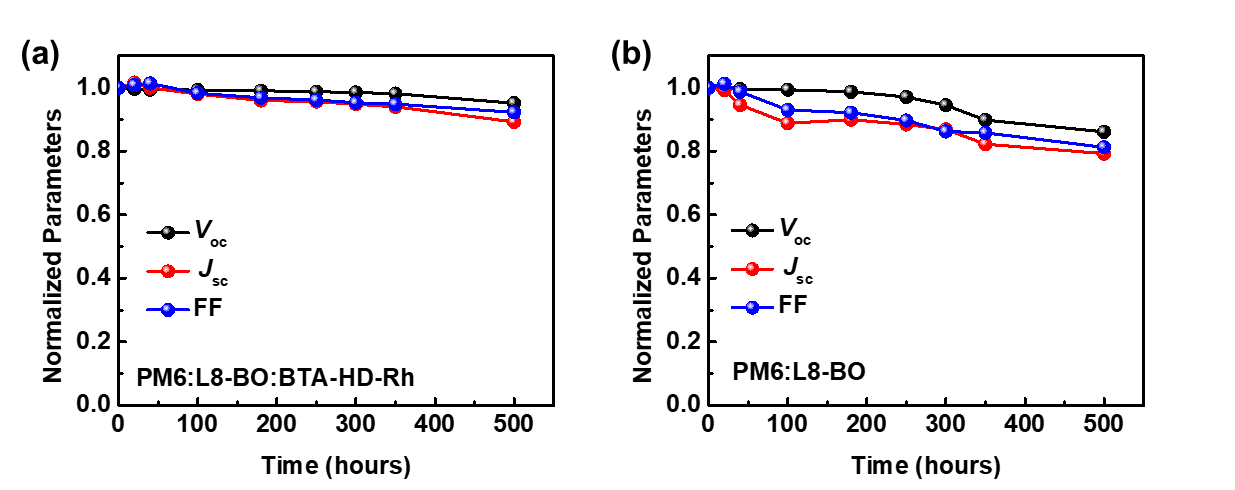
 Figure S28.** Thermal stability of sub-module optimized devices.

**
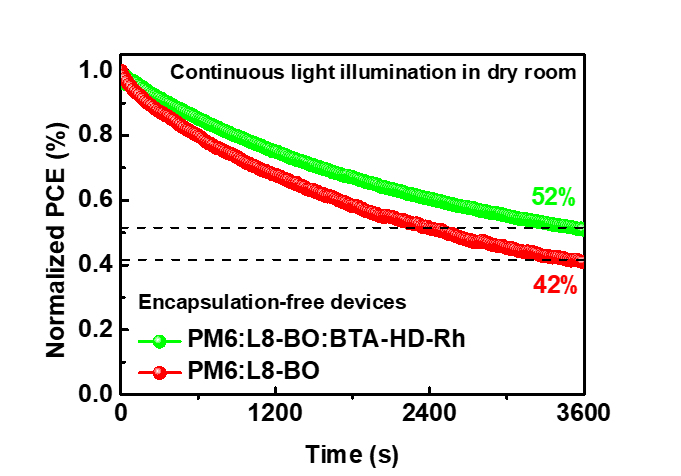
**

**Figure S29.** Photo stabilities of optimal binary and ternary devices.

**
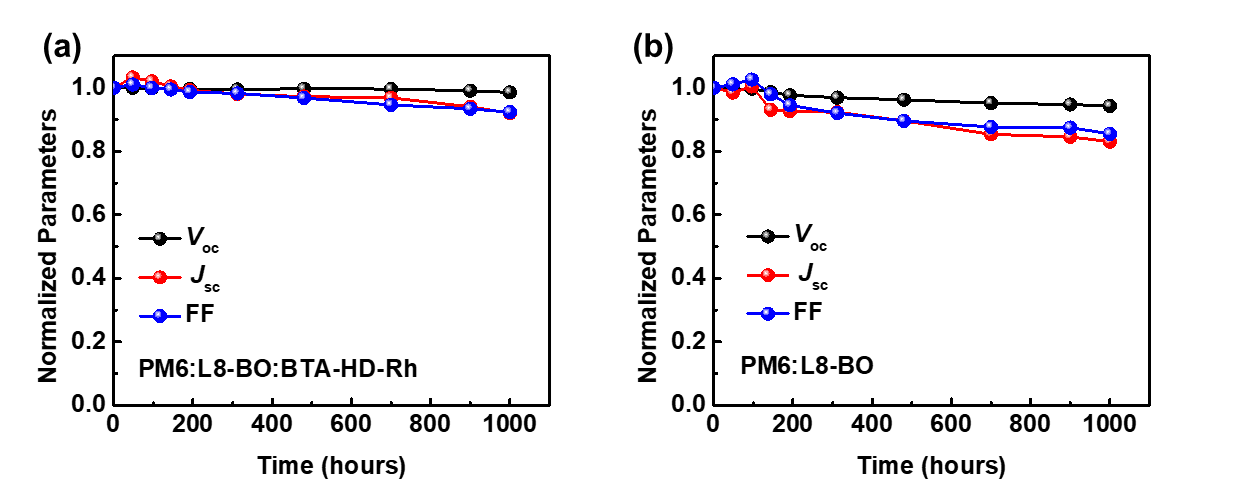
Figure S30.** Nitrogen atmosphere storage of small-area optimized devices.

**
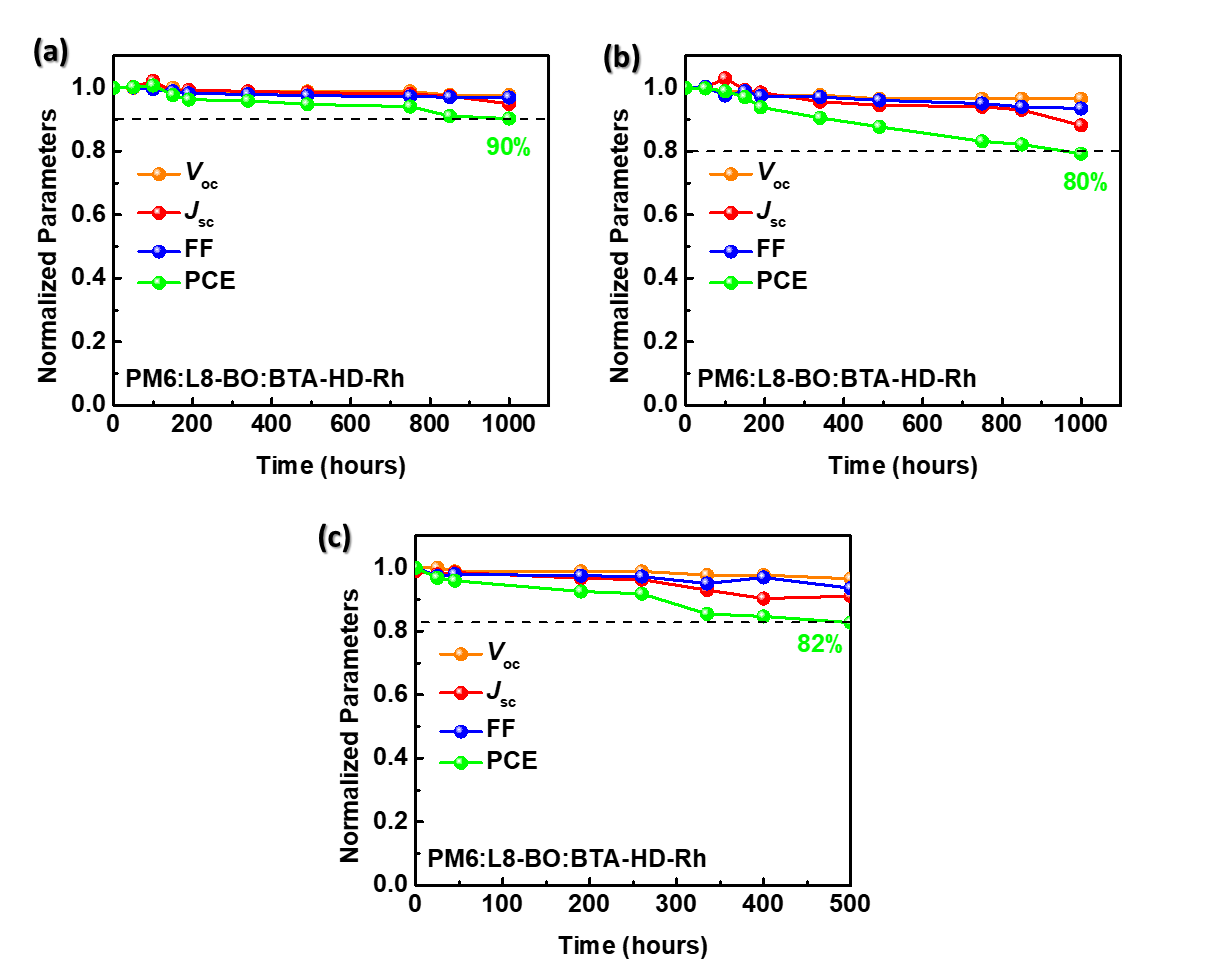
Figure S31.** Device stabilities of optimal ternary small-area devices. Air stabilities (a) 25 °C, RH: 30%; (b) 22 °C, RH: 60%, and (b) thermal stabilities (80°C in dry room).

**Table S1.** The recent reported photovoltaic parameters of solar sub-module with active areas >20 cm^2^.

| References | Area  (cm^2^) | *V*_OC_ (V) | *J*_SC_ (mA cm^–2^) | FF (%) | PCE (%) | Solvent |
| --- | --- | --- | --- | --- | --- | --- |
| **This work** | **55** | **9.49** | **2.50** | **67.36** | **16.03** | ***O*-XY:CS_2_** |
| [1] Our lab | 55 | 9.36 | 2.35 | 63.43 | 13.88 | *o-*xylene |
| [2] Our lab | 55 | 8.81 | 2.32 | 61.05 | 12.20 | CB:CF |
| [7] | 58.5 | 8.80 | 1.71 | 60 | 9.03 | Chlorobenzene |
| [8] | 58.5 | 8.61 | 2.43 | 67.10 | 14.01 | Chloroform |
| [9] | 58.5 | 8.16 | 2.21 | 63.7 | 11.28 | Chloroform |
| [10] | 54.45 | 7.97 | 1.34 | 61 | 6.61 | Xylene |
| [11] | 54.45 | 9.32 | 1.73 | 71.9 | 11.60 | *o*-xylene |
| [12] | 50 | 8.74 | 2.60 | 58 | 13.12 | Chloroform |
| [13] | 36 | 10.02 | 2.01 | 70.8 | 14.26 | Paraxylene |
| [14] | 36 | 9.90 | 1.79 | 70.53 | 13.47 | Chloroform |
| [15] | 31.50 | 4.27 | 4.65 | 62.61 | 12.44 | Xylene |
| [16] | 25.21 | 8.92 | 2.19 | 72 | 14.07 | CF:CB |
| [17] | 25.2 | 6.01 | 3.42 | 70.17 | 14.42 | o-xylene |
| [18] | 21 | 6.1 | 3.39 | 74.6 | 15.40 | o-xy:CS_2_ |
| [24] | 15.64 | 7.14 | 2.92 | 75.31 | 15.69 | Toluene |
| [25] | 15.03 | 6.85 | 3.24 | 71.84 | 15.97 | Toluene |

**Table S2.** Optical, thermal and electrochemical properties of new NFA.

| NFA | λ_max_^a)^  [nm] | λ_max_^b)^ [nm] | E_g_^optc)^ [eV] | T_d_^d)^  [ºC] | HOMO ^e)^ [eV] | LUMO ^f)^  [eV] |
| --- | --- | --- | --- | --- | --- | --- |
| BTA-HD-Rh | 520, 620 | 583, 637 | 1.78 | 333 | -5.21 | -3.34 |
| BTA-ERh^g)^ | 620 | 660 | 1.68 | 373 | -5.47 | -3.63 |

^a)^Measured in chloroform solution with a concentration of 10^-5^ mol L^-1^. ^b)^Measured in neat film. ^c)^Optical bandgap (*E*_g_^opt^), calculated from the onset of UV-vis spectrum, *E*_g_^opt^ = 1240/λ. ^d)^Temperature with 5% mass loss was measured by TGA with a heating rate of 10 ºC min^-1^ under N_2_. ^e)^HOMO value was determined by the onset of CV measurement. ^f)^LUMO value was calculated from HOMO value, LUMO=HOMO+*E*_g_^opt^. ^g)^Our model NFA (*ACS Appl. Mater. Interfaces* **2023**, *15*, 19307).

**Table S3.** Photovoltaic parameters of optimization process of BTA-HD-Rh blended sub-modules (RH: 30 to 45%).

| Thickness  (nm) | Coat.  Cond. | Annealing  (^o^C) | X | Voc (V) | FF (%) | Jsc (mA/cm^2^) | PCE  (%) |
| --- | --- | --- | --- | --- | --- | --- | --- |
| 86 | 12/10 uL | 100 °C/20 min | 1 | 9.09 | 2.55 | 60.43 | 14.03 |
| 120 | 14/13 uL | 100 °C/15 min | 2 | 9.20 | 2.40 | 61.93 | 13.72 |
| 150 | 15/12 uL | 100 °C/15 min | 3 | 9.26 | 2.46 | 61.19 | 13.97 |
| 170 | 15/13 uL | 120 °C/20 min | 4 | 9.49 | 2.50 | 67.36 | 16.03 |
| 200 | 15/10 uL | 130 °C/20 min | 5 | 9.31 | 2.54 | 56.84 | 13.48 |
| 248 | 15/12 uL | 100 °C/20 min | 6 | 9.13 | 2.57 | 55.78 | 13.12 |
| 170 | 15/12 uL | - | 7 | 9.36 | 2.49 | 61.21 | 14.32 |
| 170 | 15/13 uL | 120 °C/10 min | 8 | 9.34 | 2.59 | 62.70 | 15.17 |
| 170 | 15/13 uL | 120 °C/30 min | 9 | 9.28 | 2.51 | 66.40 | 15.51 |
| 170 | 15/10 uL | - | 10 | 9.25 | 2.44 | 60.49 | 13.69 |

X: Devices

Device architecture: ITO-glass/ZnO-NPs/active layer/MoO_3_/Ag. *Active layer:* PM6:L8-BO:BTA-HD-Rh. Solvent: (*O*-XY:CS_2_, v/v = 0.6:0.4)

**Table S4.** Sub-module photovoltaic parameters for ternary active layer with various blend concentrations under simulated solar illumination (AM 1.5G,100 mW/cm^2^).

| Blend concentrations | *V*oc (V) | *J*sc (mA/cm^2^) | FF (%) | PCE (%) |
| --- | --- | --- | --- | --- |
| 12 mg/ml | 9.28 | 2.52 | 62.87 | 14.80 |
| 15 mg/ml | 9.49 | 2.50 | 67.36 | 16.03 |
| 18 mg/ml | 9.27 | 2.64 | 62.14 | 15.17 |
| 22 mg/ml | 9.38 | 2.65 | 59.83 | 14.88 |
| 25 mg/ml | 9.22 | 2.52 | 52.69 | 12.27 |

Active layer: PM6:L8-BO:BTA-HD-Rh (1:1:0.2, wt ratio). Solvent: (*O*-XY:CS_2_, v/v = 0.6:0.4)

**Table S5.** Sub-module photovoltaic parameters for binary and ternary devices under simulated solar illumination (AM 1.5G,100 mW/cm^2^).

| PM6:L8-BO:BTA-HD-Rh (wt ratio) | *V*oc (V) | *J*sc (mA/cm^2^) | FF (%) | PCE (%) |
| --- | --- | --- | --- | --- |
| 1:1.2:0 | 9.24 | 2.59 | 60.48 | 14.51 |
| 1:1:0.2 | 9.49 | 2.50 | 67.36 | 16.03 |
| 1:0.8:0.4 | 9.43 | 2.51 | 65.45 | 15.44 |
| 1:0.6:0.6 | 9.41 | 2.62 | 63.98 | 15.82 |
| 1:0.4:0.8 | 9.34 | 2.59 | 62.70 | 15.17 |
| 1:0:1.2 | 8.76 | 1.50 | 57.56 | 7.47 |

Solvent: (*O*-XY:CS_2_, v/v = 0.6:0.4)

**Table S6.** Detailed sub-module photovoltaic parameters of processed by environmentally benign non-halogen solvents with different NFAs under an illumination of AM 1.5G at 100 mW cm^‒2^.

| Active layers^a)^ | *V*_oc_ [V] | *J*_sc_ [mA/cm^2^] | FF [%] | PCE^b)^ [%] |
| --- | --- | --- | --- | --- |
| PM6:L8-BO:BTA-HD-Rh | 9.49 | 2.50 | 67.36 | 16.03 |
| PM6:Y7-BO:BTA-HD-Rh | 9.43 | 2.48 | 66.60 | 15.62 |
| PM6:Y6-BO:BTA-HD-Rh | 9.42 | 2.44 | 66.47 | 15.31 |
| PM6:BTP-eC9:BTA-HD-Rh | 9.40 | 2.59 | 63.69 | 15.52 |

^a)^Dissolved in *O*-XY:CS_2_ (v/v = 0.6:0.4) solvents and their blend films were pre-annealed at 120 °C/20 min. ^b)^An average of 15 sub-modules were tested.

**Table S7.** Detailed small-area devices photovoltaic parameters of processed by environmentally benign non-halogen solvents under an illumination of AM 1.5G at 100 mW cm^‒2^.

| Active layers^a)^ | *V*_oc_ [V] | *J*_sc_ [mA/cm^2^] | FF [%] | PCE^b)^ [%] |
| --- | --- | --- | --- | --- |
| PM6:L8-BO | 0.85 | 26.12 | 71.26 | 16.04 |
| PM6:L8-BO:BTA-HD-Rh | 0.86 | 27.54 | 75.08 | 17.80 |
| PM6:BTA-HD-Rh | 0.82 | 18.45 | 55.30 | 8.46 |

^a)^Dissolved in *O*-XY:CS_2_ (v/v = 0.8:0.2) solvents and their blend films were pre-annealed at 100 °C/10 min. ^b)^An average of 20 devices were tested.

**Table S8.** Small-area ternary blend devices optimization under simulated solar illumination (AM 1.5G,100 mW/cm^2^).

| PM6:L8-BO:BTA-HD-Rh | *V*oc (V) | | *J*sc (mA/cm^2^) | FF (%) | PCE (%) |
| --- | --- | --- | --- | --- | --- |
| (wt ratio) | |  |  |  |  |
| 1:1.2:0 | | 0.85 | 26.12 | 71.26 | 16.04 |
| 1:1:0.2 | | 0.86 | 27.54 | 75.08 | 17.80 |
| 1:0.8:0.4 | | 0.86 | 26.73 | 73.48 | 17.04 |
| 1:0.6:0.8 | | 0.83 | 26.79 | 71.60 | 16.11 |
| 1:1.2 | | 0.82 | 18.45 | 55.30 | 8.46 |
| PM6:L8-BO:BTA-HD-Rh (Annealing) | |  |  |  |  |
| 80 °C/10 min | | 0.86 | 27.91 | 69.16 | 16.73 |
| 100 °C/10 min | | 0.86 | 27.54 | 75.08 | 17.80 |
| 120 °C/10 min | | 0.86 | 26.87 | 72.83 | 16.87 |
| 150 °C/10 min | | 0.86 | 25.06 | 69.33 | 15.05 |
| PM6:L8-BO:BTA-HD-Rh (Thickness) | |  |  |  |  |
| 90 nm | | 0.87 | 24.96 | 72.79 | 15.94 |
| 100 nm | | 0.86 | 26.15 | 73.99 | 16.74 |
| 120 nm | | 0.86 | 27.54 | 75.08 | 17.80 |
| 150 nm | | 0.85 | 27.86 | 71.74 | 17.10 |
| 200 nm | | 0.85 | 27.19 | 71.89 | 16.65 |

Solvent: (*O*-XY:CS_2_, v/v = 0.8:0.2). (RH: 30 to 45%).

**Table S9.** The recent reported photovoltaic parameters of air-processed small-area devices with non-halogen solvents.

| References | *V*_OC_ (V) | *J*_SC_ (mA cm^–2^) | FF (%) | PCE (%) | Solvent |
| --- | --- | --- | --- | --- | --- |
| **This work** | **0.86** | **27.54** | **75.08** | **17.80** | ***O*-XY:CS_2_** |
| [1] Our lab | 0.86 | 28.17 | 74.32 | 17.96 | *o*-xylene |
| [19] | 0.84 | 26.77 | 76.33 | 17.15 | *o*-xylene |
| [15] | 0.85 | 26.68 | 76.52 | 17.38 | *o*-xyl+DPE |
| [19] | 0.85 | 27.23 | 73.96 | 17.12 | Tol+DIO |
| [13] | 0.85 | 27.12 | 75.75 | 17.41 | p-xyl+CN |
| [21] | 0.85 | 25.52 | 75.10 | 16.33 | TMB |
| [22] | 0.88 | 17.62 | 75.78 | 11.76 | MTHF |
| [23] | 0.95 | 16.80 | 66.10 | 11.00 | Toluene |

**Table S10.** Hole and electron mobility of optimized devices.

| Active layers | *µ*_h_^a)^  (cm^2^ V^-1^ S^-1^) ×10^-3^ | *µ*_e_^a)^  (cm^2^ V^-1^ S^-1^) ×10^-3^ | *µ*_h_/*µ*_e_ |
| --- | --- | --- | --- |
| BTA-Erh^b)^ | - | 3.50 | - |
| BTA-HD-Rh | - | 3.72 | - |
| L8-BO:BTA-HD-Rh | - | 3.81 | - |
| PM6:L8-BO | 2.57 | 2.68 | 0.95 |
| PM6:L8-BO:BTA-HD-Rh | 3.65 | 3.43 | 1.06 |

^a)^Measured by space charge limited current (SCLC) method.

^b)^Our model NFA *(ACS Appl. Mater. Interfaces* ***2023****, 15, 19307).*

*Hole only (µ*_h_*) device architecture:* ITO-glass/PEDOT:PSS/active layer/MoO_3_/Ag.

*Electron only (µ*_e_*) device architecture:* ITO-glass/ZnO/PFN-Br/active layer/PDINO/Ag.

**Table S11.** Contact angle and surface energy (γ_s_) studies for PM6, L8-BO, BTA-HD-Rh, and L8-BO:BTA-HD-Rh films.

| Films | Θ (H_2_O)  (degree) | Θ (CH_2_I_2_)  (degree) | γ^d a^  (mJ m^-2^) | γ^p b^  (mJ m^-2^) | γ_s_ = ( γ^d a^+γ^p b^)  (mJ m^-2^) |
| --- | --- | --- | --- | --- | --- |
| PM6 | 108.07±0.27 | 57.20±0.22 | 28.8 | 0.1 | 28.9 |
| L8-BO | 96.18±0.17 | 53.23±0.20 | 31.7 | 0.7 | 32.5 |
| BTA-HD-Rh | 98.14±0.32 | 55.72±0.36 | 30.5 | 0.6 | 31.1 |
| L8-BO:BTA-HD-Rh | 97.26±0.23 | 56.42±0.34 | 32.1 | 1.0 | 33.2 |

**γ_s_**: surface energy (dispersion liquid + polar liquid). **γ^d a^**:Dispersion of surface tension. **γ^p b^**:Polar component of surface tension.

[The surface energies were calculated using the Owens–Wendt (O–W) geometric method.]^[5,6]^

**Table S12.** Optimized devices relative parameters obtained from *J*_ph_-*V*_eff_ curves.

| Active layers | *J*_ph_  (mA/cm^2^) | *J*_sat_  (mA/cm^2^) | *G*_max_  (m^-3^ s^-1^) (×10^28^) | *P (E, T)*^a)^  (%) |
| --- | --- | --- | --- | --- |
| PM6:L8-BO:BTA-HD-Rh | 26.87 | 27.68 | 1.39 | 97.07 |
| PM6:L8-BO | 26.12 | 28.36 | 1.25 | 92.10 |

^a)^Under short-circuit condition, *V_appl_* = 0, *Device architecture:* ITO-glass/ZnO/PFN-Br/active layer/MoO_3_/Ag.

**References**

1. T. Gokulnath, H. Kim, J. Lee, B. H. Cho, H.-Y. Park, J. Jesung, Y. Y. Kim, K. Kranthiraja, J. Yoon, Sung-Ho Jin, *Adv. Energy Mater.* **2023**, 2302538.
2. T. Gokulnath, J. Kim, H. Kim, J. Park, D. Song, H.-Y. Park, R. Kumaresan, Y. Y. Kim, J. Yoon, S.-H. Jin, *ACS Appl. Mater. Interfaces* **2023**, *15*, 19307.
3. (a) J. Fu, P. W. K. Fong, H. Liu, C.-S. Huang, X. Lu, S. Lu, M. Abdelsamie, T. Kodalle, C. M. Sutter-Fella, Y. Yang, G. Li, *Nat Commun* **2023**, 14, 1760; (b) Y. Y. Kim, J. Kim, N. Kim, https://doi.org/10.5281/zenodo.7042272 [pGIXS: PLS-II 3C SAXS beamline data plot program with MATLAB (0.1 version). Zenodo.]
4. H. S. Park, Y. W. Han, H. S. Lee, S. J. Jeon, D. K. Moon, *ACS Appl. Energy Mater.* **2020**, *3*, 3745
5. T. Gokulnath, S.S. Reddy, H.-Y. Park, J. Kim, J. Kim, M. Song, J. Yoon, S.-H. Jin, *Sol. RRL,* **2021**, 2000787
6. K. Kranthiraja, U. K. Aryal, V. G. Sree, K. Gunasekar, C. Lee, M. Kim, B. J. Kim, M. Song, S.-H. Jin, *ACS Appl. Mater. Interfaces* **2018**, *10*, 13748.
7. S. H. Park, S. Park, S. Lee, J. Kim, H. Ahn, B. J. Kim, B. Chae, H. J. Son, *Nano Energy,* **2020**, *77,* 105147.
8. S. Yoon, S. Park, S. H. Park, S. Nah, S. Lee, J.-W. Lee, H. Ahn, H. Yu, E.-Y. Shin, B. J. Kim, B. K. Min, J. H. Noh, H. J. Son, *Joule,* **2022**, *6,* 1-17.
9. P. Gopikrishna, J. Rhee, S. Yoon, D. Um, H. Jin, Y. Jun, H. Choi, H. J. Son, B. Kim, *Adv. Funct. Mater.* **2023**, 2305541.
10. S. Rasool, D. V. Vu, C. E. Song, H. K. Lee, S. K. Lee, J.-C. Lee, S.-J. Moon, W. S. Shin, *Adv. Energy Mater.,* **2019**, *9*, 1900168.
11. Z. U. Rehman, M. Haris, S. U. Ryu, M. Jahankhan, C. E. Song, H. K. Lee, S. K. Lee, W. S. Shin, T. Park, J.-C. Lee, *Adv. Sci.* **2023**, 2302376.
12. M. Jahandar, A. Prasetio, C. Lee, H. Kim, A. R. Kim, J. Heo, Y. Kim, S. Kim, D. C. Lim, *Chem. Eng. J.,* **2022**, *448,* 137555.
13. H. Chen, R. Zhang, X. Chen, G. Zeng, L. Kobera, S. Abbrent, B. Zhang, W. Chen, G. Xu, J. Oh, S. H. Kang, S. Chen, C. Yang, J. Brus, J. Hou, F. Gao, Y. Li, Y. Li, *Nat. Energy* **2021**, *6,* 1045.
14. B. Zhang, F. Yang, S. Chen, H. Chen, G. Zeng, Y. Shen, Y. Li, Y. Li, *Adv. Funct. Mater.* **2022**, *32,* 2202011.
15. S. Rasool, J. W. Kim, H. W. Cho, Y.-J. Kim, D. C. Lee, C. B. Park, W. Lee, O.-H. Kwon, S. Cho, J. Y. Kim, *Adv. Energy Mater.* **2022**, 2203452.
16. X. Dong, Y. Jiang, L. Sun, F. Qin, X. Zhou, X. Lu, W. Wang, Y. Zho, *Adv. Funct. Mater.* **2022**, *32*, 2110209.
17. S. Zhang, H. Chen, P. Wang, S. Li, Z. Li, Y. Huang, J. Liu, Z. Yao, C. Li, X. Wan, Y. Chen, *Sol. RRL* **2023**, *7*, 2300029.
18. Z. Zhong, S. Chen, J. Zhao, J. Xie, K. Zhang, T. Jia, C. Zhu, J. Jing, Y. Liang, L. Hong, S. Zhu, D. Ma, F. Huang, *Adv. Energy Mater.* **2023**, 2302273.
19. J. Xue, H. B. Naveed, H. Zhao, B. Lin, Y. Wang, Q. Zhu, B. Wu, Z. Bi, X. Zhou, C. Zhao, K. Zhou, W. Ma, *J. Mater. Chem. A* **2022**, *10,* 13439.
20. Y. Zhang, K. Liu, J. Huang, X. Xia, J. Cao, G. Zhao, P. W. K. Fong, Y. Zhu, F. Yan, Y. Yang, X. Lu, G. Li, *Nat. Commun.* **2021**, *12,* 4815.
21. R. Ma, T. Yang, Y. Xiao, T. Liu, G. Zhang, Z. Luo, G. Li, X. Lu, H. Yan, B. Tang, *Energy Environ. Mater.* **2021**, 1.
22. L. Zhu, W. Zhong, C. Qiu, B. Lyu, Z. Zhou, M. Zhang, J. Song, J. Xu, J. Wang, J. Ali, W. Feng, Z. Shi, X. Gu, L. Ying, Y. Zhang, F. Liu, *Adv. Mater.* **2019**, *31*, 1902899.
23. L. Ye, Y. Xiong, Q. Zhang, S. Li, C. Wang, Z. Jiang, J. Hou, W. You, H. Ade, *Adv. Mater.* **2018**, *30,* 1705485.
24. B. Zhang, W. Chen, H. Chen, G. Zeng, R. Zhang, H. Li, Y. Wang, X. Gu, W. Sun, H. Gu, F. Gao, Y. Li, Y. Li, *Energy Environ. Sci.,* **2024**, *17,* 2935.
25. H. Chen, W. Sun, R. Zhang, Y. Huang, B. Zhang, G. Zeng, J. Ding, W. Chen, F. Gao, Y. Li, Y. Li, *Adv. Mater.* **2024**, 2402350.
